# Productive, physiological, and biochemical responses of elephant grass ‘BRS Capiaçu’ (*Pennisetum purpureum* Schumach.) under different water regimes with brackish water in a semi-arid environment

**DOI:** 10.1007/s12298-026-01792-6

**Published:** 2026-07-21

**Authors:** Lara Rosa de Lima-Silva, Lady Daiane Costa de Sousa Martins, Alexandre Maniçoba da Rosa Ferraz Jardim, Wagner Martins dos Santos, Pedro Paulo Santos de Souza, Luciana Sandra Bastos de Souza, Cleber Pereira Alves, Agda Raiany Mota dos Santos, Ênio Farias de França e Silva, Hugo Rafael Bentzen Santos, Wilma Roberta dos Santos, Carlos André Alves de Souza, Adriano Nascimento Simões, Sérgio Luiz Ferreira-Silva, Elania Freire da Silva, José Francisco da Cruz Neto, Xuguang Tang, João L. M. P. de Lima, Thieres George Freire da Silva

**Affiliations:** 1 Postgraduate Program in Plant Production, Academic Unit of Serra Talhada, Federal Rural University of Pernambuco, Serra Talhada, Pernambuco 56909-535 Brazil; 2 Institute of Remote Sensing and Geosciences, Hangzhou Normal University, Hangzhou, 311121 China; 3 Department of Agricultural Engineering, Federal Rural University of Pernambuco, Recife, Pernambuco 52171-900 Brazil; 4 Department of Biodiversity, Institute of Biosciences, São Paulo State University—UNESP, Rio Claro, São Paulo 13506-900 Brazil; 5 Department of Botany, Federal University of Pernambuco, Recife, Pernambuco 50670-901 Brazil; 6 Department of Agricultural and Forestry Sciences, Federal Rural University of the Semi- Arid, Mossoró, Rio Grande do Norte 59625-900 Brazil; 7 Department of Agronomy, State University of Maranhão, Balsas, Maranhão Brazil; 8 MARE—Marine and Environmental Sciences Centre, ARNET—Aquatic Research Network, Department of Civil Engineering, Faculty of Sciences and Technology, University of Coimbra, Rua Luís Reis Santos, Pólo II—Universidade de Coimbra, Coimbra, 3030-788 Portugal

**Keywords:** Photosynthesis, Forage plants, Antioxidant enzymes, Reference evapotranspiration, Chlorophyll *a* fluorescence

## Abstract

The seasonality of forage production is one of the main challenges in semi-arid regions, where environmental conditions limit agricultural productivity. One promising option is the use of elephant grass ‘BRS Capiaçu’, together with deficit irrigation. This study evaluated the impact of different irrigation depths with brackish water (50%, 75%, 100% and 125% of the reference evapotranspiration – ET_0_) on the productive, physiological, photochemical and biochemical aspects of ‘BRS Capiaçu’ over two cycles. Irrigation with 50% of the ET_0_ resulted in lower rates of net photosynthesis (P_*n*_), with a reduction in photosystem II quantum yield (ΔF/Fm’), qP, NPQ and the electron transport rate (ETR), in addition to greater activity of the antioxidant enzymes superoxide dismutase (SOD), catalase (CAT) and ascorbate peroxidase (APX), indicating oxidative stress. The depths of 50% and 100% of the ET_0_ resulted in greater dry matter production, for Cycles 1 and 2, respectively, demonstrating the resilience of the crop to water stress, attributed to such mechanisms of acclimatisation as a reduction in transpiration (T_*r*_) and an increase in water use efficiency. These physiological changes were more obvious during Cycle 2, in which the crop was subjected to lower rainfall (47.8 mm), while salt stress was also more pronounced during this period, in response, the plants showed less water loss through transpiration and larger WUE_inst_. It was concluded that ‘BRS Capiaçu’ has a high capacity for acclimatisation to semi-arid conditions, and stands out as a viable alternative for mitigating the seasonality of forage production and promoting sustainable livestock farming in the region.

## Introduction

Semi-arid regions present biophysical limitations that affect agricultural production, such as high temperatures, low relative humidity, low rainfall and irregular rainfall distribution, which can lead to anything from extreme droughts to floods, due to the high interannual variability of the climate (Alves et al. 2022; Karolina et al. 2025; Singh and Chudasama 2021). Such conditions can result in seasonal forage production, which makes the food supply for livestock scarce during periods of drought (Silva et al. 2024a, b). Adopting resilient agricultural practices, such as the cultivation of adapted species and the use of supplemental irrigation, is therefore essential to mitigate the impact of the semi-arid climate on agriculture, especially to compensate for the irregular rainfall (Araujo Júnior et al. 2024; Lankford et al. 2023; Jardim et al. 2023).

Linked to the seasonality of food production and driven by the growth in population, there is a growing demand for animal products, making it essential to improve the productivity of forage crops to meet the nutritional requirements of livestock and ensure quality products (Prudente Junior 2019). With the aim of expanding the options for bulk supplements in animal diets, even under water scarcity, the Brazilian Agricultural Research Corporation (EMBRAPA) developed the ‘BRS Capiaçu’ cultivar (*Pennisetum purpureum* Schumach.), which is derived from elephant grass and characterised by its high nutritional value and high potential for forage production (Moura et al. 2024).

Despite its resilience to water deficit, its productivity is improved by the presence of water in the soil, such as in an irrigated system (Santos et al. 2023). Santos et al. (2025) evaluated the dry matter production and economic viability of forage plants in a semi-arid environment under irrigation with 100% of the reference evapotranspiration, and achieved yields of up to 60 Mg ha^− 1^ for ‘BRS Capiaçu’, in addition to a positive cost-benefit ratio, attributed to its versatility and the possibility of multiple production cycles. Due to the scarcity of good-quality water in these regions, it is important to adopt irrigation management that minimises the effects of salinity and maximises water use by the plants. It is therefore necessary to understand how the crop responds to the local water conditions (Araújo Júnior et al. 2023; Chilundo et al. 2016; Kresović et al. 2016). Understanding how plants react to water deficit, with physiological changes that affect growth and productivity, is essential for the efficient application of deficit irrigation (Silva et al. 2013; Ghannoum 2009).

Studies on these changes in plants under varying conditions of water availability, particularly water deficit, can be increasingly found in the literature (Ghannoum 2009; Silva et al. 2013). For example, when studying the anatomical, physiological and biochemical changes caused by water deficit in two genotypes of elephant grass, Araújo et al. (2010) found that, under ideal conditions of soil moisture the photosynthetic rate remains high, regardless of the cultivar. Habermann et al. (2019) investigated the effect of soil water deficit and a 2 °C increase in canopy temperature on *Panicum maximum*. The results showed that water stress, even at room temperature, reduced the net photosynthetic rate, stomatal conductance and maximum rate of Rubisco carboxylation, in addition to reducing the starch content of the leaves, compromising the quality and digestibility of the grass. Cavalcante et al. (2021), evaluating maize productivity in a semi-arid environment under four water scenarios (rainy, normal, dry and severe drought), with and without supplemental irrigation with brackish water (4.5 dS m^− 1^), found that sweet corn production was reduced in the absence of supplemental irrigation, regardless of the scenario.

However, studies evaluating the effects of different water regimes on the physiological and biochemical characteristics of forage grasses are still rare. Research on ‘BRS Capiaçu’ generally focuses on such aspects as productivity, silage yield and nutritional value (Monção et al. 2019; Pereira et al. 2016; Retore et al. 2021). The aim of this study was to evaluate the effect of different irrigation depths with brackish water on the productive, physiological, photochemical and biochemical parameters of elephant grass ‘BRS Capiaçu’.

## Materials and methods

### Study area

**Fig. 1:**
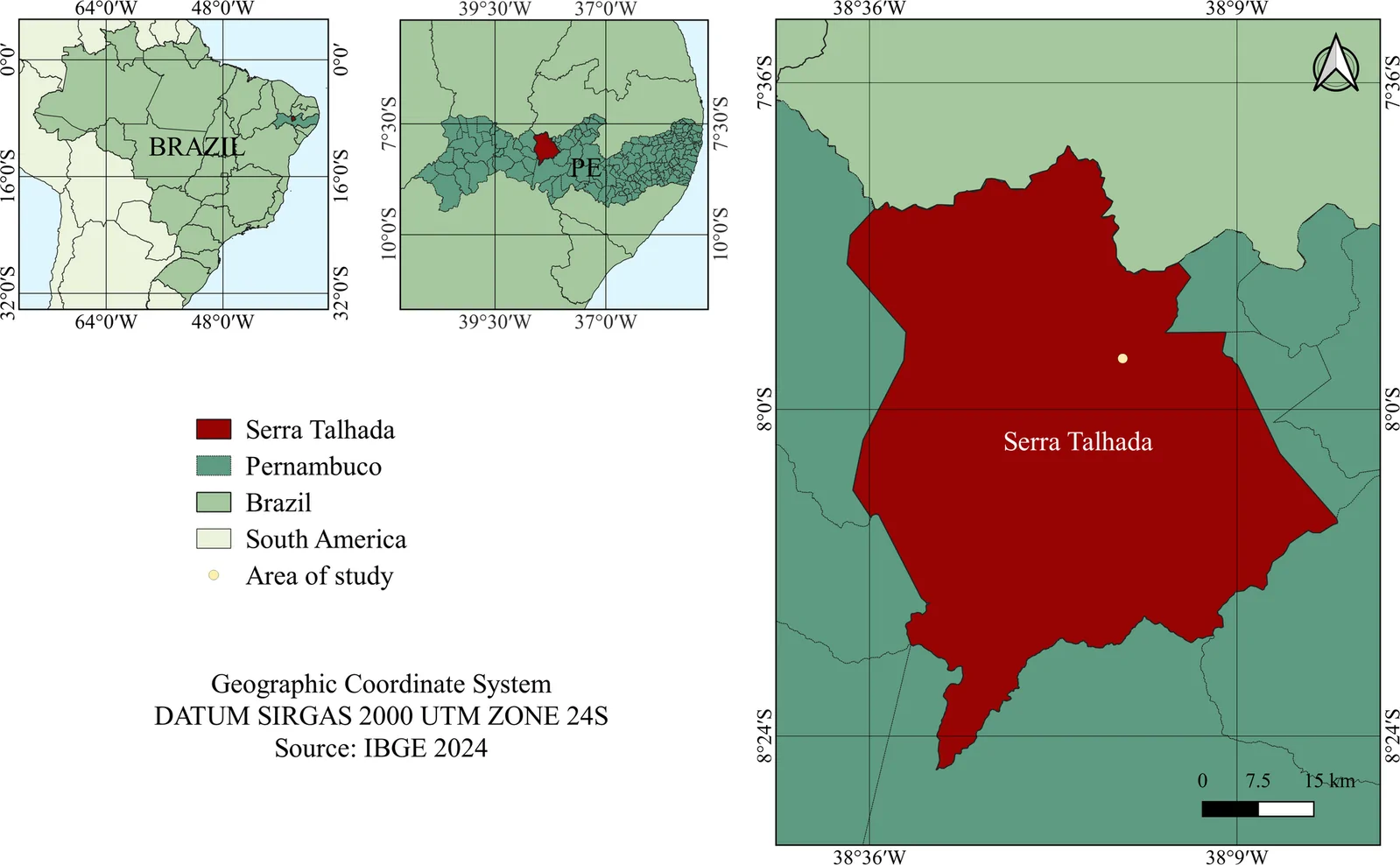
Location of the experimental area, in the district of Serra Talhada, Pernambuco (PE), Brazil

The experiment was conducted at the International Reference Centre for Agrometeorological Studies of the Cactus and other Forage Plants at the Serra Talhada Academic Unit (UAST/UFRPE), located at 7º56’20” S and 38º17’31” W at an altitude of 499 m, in the district of Serra Talhada, in the Sertão Mesoregion of the State of Pernambuco (Brazil), 415 km from the state capital, Recife (Fig. 1).

According to the Köppen climate classification, the climate in the region is semi-arid type BShw (hot-dry semi-arid, with rainy periods during the summer and winter). It is characterised by low rainfall, high air temperatures and an atmospheric demand that exceeds the rainfall (Alvares et al. 2013). The air temperature ranges from 20.1 to 32.9 °C, with an average relative humidity of 63%, average annual rainfall of 642 mm and an atmospheric demand greater than 1800 mm yr^− 1^, generating a water deficit of more than 1100 mm yr^− 1^ (Pereira et al. 2015).

### Experimental design and treatments

The experiment was conducted over a total area of 320 m^2^ using ‘BRS Capiaçu’ grass, and organised in a randomised block design (RBD) with four replications. Four levels of irrigation were evaluated, corresponding to 50%, 75%, 100% and 125% of the reference evapotranspiration (ET_0_), giving a total of 16 plots of 5 × 4 m. Each plot consisted of four rows of plants, two of which were working rows and two were borders. Two cycles of ‘BRS Capiaçu’ were evaluated: the first from 14 November 2023 to 20 March 2024 (spring-summer), and the second from 27 June 2024 to 16 October 2024 (winter-spring).

### Experimental procedure

The soil in the experimental area is classified as a typical Eutrophic Haplic Cambisol as per the Brazilian System of Soil Classification (Santos et al. 2018). The physical and chemical properties of the soil are shown in Table 1.

**Table 1:** Physical and chemical properties of the soil in the experimental area

Depth	ρd		Ø		Sand		Silt		Clay	
Physical properties										
cm	kg dm^-3^		%		g kg^-1^					
0–20	1.45		42.27		828.6		148.3		23.2	
20–40	1.34		46.76		795.4		160.1		44.6	

Depth	EC	pH	OC	P	K^+^	Na^+^	Ca^2+^	Mg^2+^	CEC	V
Chemical properties										
cm	dS m^-1^		g kg^-1^	mg dm^-3^	cmol_c_ dm^-3^					%
0–20	0.33	6.0	4.6	168.9	13.8	1.09	3.5	1.90	20.9	97.2
20–40	0.24	6.3	3.0	154.1	11.8	1.47	2.9	1.75	18.3	98.7
ρd: soil bulk density. Ø: total porosity. EC: electrical conductivity of the saturation extract. OC: organic carbon. CEC: cation exchange capacity. V: base saturation.										

Based on the soil analysis and fertiliser recommendations for the state of Pernambuco (Cavalcanti et al. 2008), topdressing was applied for plant growth with 40, 60 and 80 kg ha^− 1^ N, P and K^+^, respectively, using urea, single superphosphate and potassium chloride as the source. Maintenance fertiliser was applied at doses of 40, 100 and 120 kg ha^− 1^ of N, P and K^+^, respectively, using the same sources as for the topdressing. Soil preparation included mechanised ploughing, harrowing, and furrowing. As ‘BRS Capiaçu’ propagates vegetatively, planting was carried out using vigorous stems in four rows of 5.0 linear metres spaced 1.0 m apart in each plot. To maintain a uniform stand, new stems were planted to fill any gaps. Phytosanitary management was carried out as needed to control invasive plants, pests and diseases.

The irrigation water, which came from an artesian well, was classified as C3S1 (high salinity), as per Richards (1954), with an average electrical conductivity of 1.55 dS m^− 1^, a Na⁺ concentration of 168.66 mg L^− 1^, a K⁺ concentration of 28.17 mg L^− 1^ and a pH of 6.84. Irrigation was carried out using a drip system with emitters spaced 0.20 m apart, at a flow rate of 1.35 L h^− 1^, a pressure of 100 kPa, an application uniformity coefficient (CUC) of 87% and an application intensity of 18 mm h^− 1^. Irrigation took place on Mondays, Wednesdays, and Fridays, based on the reference evapotranspiration (ET_0_) as estimated by the Penman-Monteith FAO-56 method (Allen 1998):

$$\:\mathrm{E}{\mathrm{T}}_{0}=\frac{0.408\varDelta\:\left(\mathrm{R}\mathrm{n}-\mathrm{G}\right)+{\upgamma\:}\:\left(\frac{900}{{\mathrm{t}}_{\mathrm{a}\mathrm{v}\mathrm{e}}+273}\right){\mathrm{u}}_{2}\:({\mathrm{e}}_{\mathrm{s}}-{\mathrm{e}}_{\mathrm{a}})}{\varDelta\:+\:{\upgamma\:}\:(1+0.34{\mathrm{u}}_{2})}$$

where ET_0_ is the reference evapotranspiration (mm day^− 1^); Rn is the net radiation (MJ m^− 2^ day^− 1^); G is the soil heat flux (MJ m^− 2^ day^− 1^); γ is the psychrometric constant (kPa ºC^− 1^); t_ave_ is the average air temperature (°C); u_2_ is the wind speed (m s^− 1^); e_s_ - e_a_ is the air saturation deficit (kPa); *∆* is the slope of the water vapour pressure curve (kPa ºC^− 1^).

**Fig. 2:**
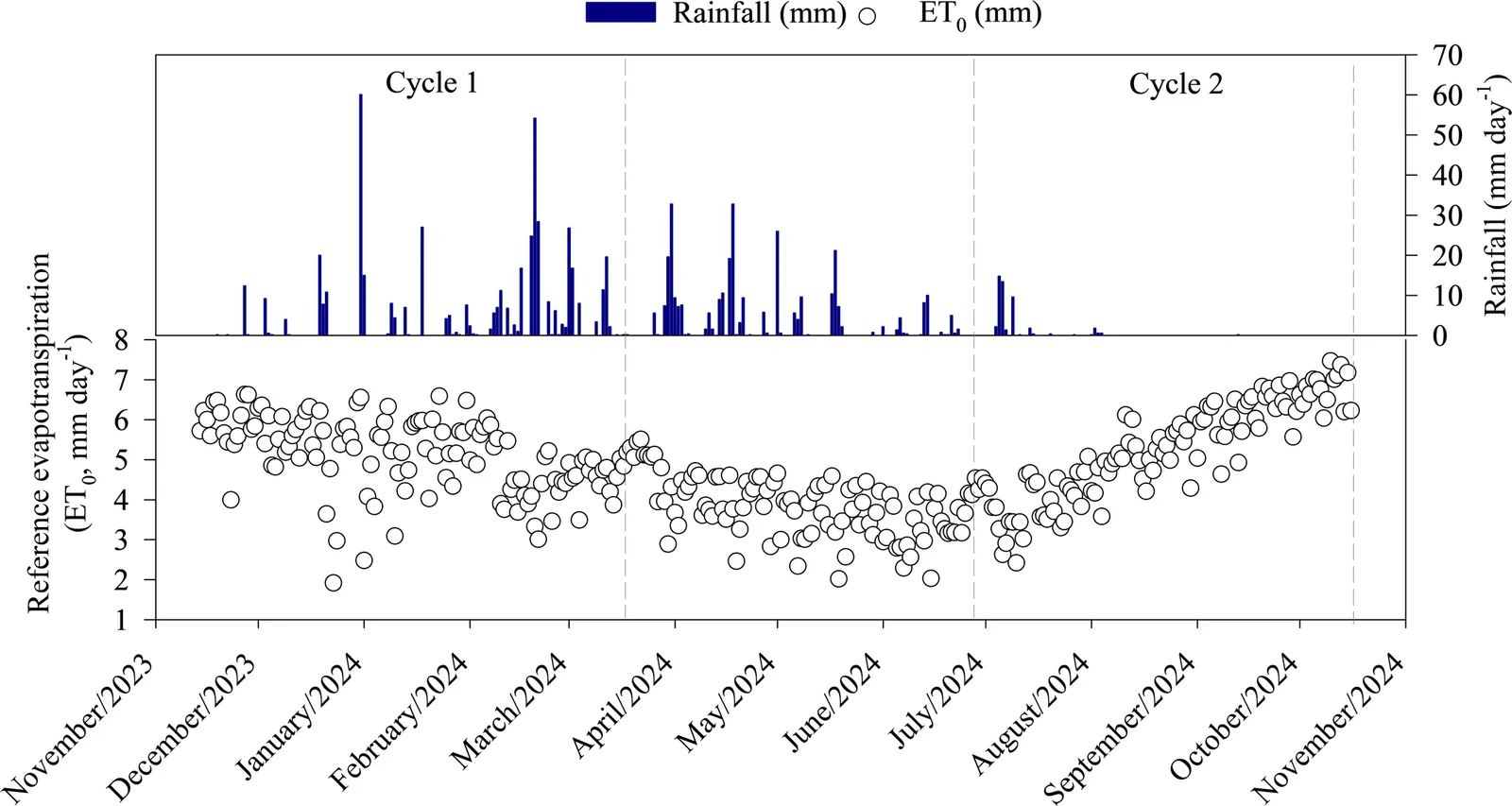
Climate conditions in the district of Serra Talhada, Pernambuco (Brazil), during the experimental period

To calculate the environmental conditions during the experimental period, daily weather data were obtained from an automatic station of the Brazilian National Institute of Meteorology (INMET), located 20 m from the experimental area. According to the records, the total rainfall volume was 477.68 mm during the first cycle and only 47.8 mm during the second (Fig. 2). Cumulative evapotranspiration reached 654.29 mm during the first cycle and 587.36 mm during the second, indicating a more severe water deficit during the second period (Fig. 2).

### Variables under analysis

#### Measuring gas exchange

The gas exchange parameters were determined from 08:00 to 11:00 using the CI-340 infrared gas analyser (CID Bio-Science Ultra-Light Portable Photosynthesis System). A 6.5 cm^2^ chamber was used, operating in an open system, at an air flow rate of 0.30 L min^− 1^, a pressure of 93.77 kPa and a mass flow rate of 0.13 mol m^− 2^ s^− 1^. Measurements were taken on the third fully expanded leaf measured from the apex toward the base of the plant, to determine the following parameters: (*i*) transpiration (T_*r*_, mmol m^− 2^ s^− 1^), (*ii*) net photosynthetic rate (P_*n*_, µmol m^− 2^ s^− 1^), (*iii*) stomatal conductance (g_*s*_, mmol m^− 2^ s^− 1^) and (*iv*) instantaneous water use efficiency (WUE_inst_ = P_*n*_/T_*r*_).

#### Chlorophyll fluorescence

The chlorophyll fluorescence parameters were measured from 09:00 to 12:00 using a Mini-PAM II portable pulse-modulated fluorometer (Heinz Walz, Effeltrich, Germany). Assessments were taken on the third fully expanded leaf measured from the apex toward the base of the plant. Before taking the readings, the leaves underwent a dark pre-adaptation period of 50 min, using support clips attached around the leaves (Jardim et al. 2021). After adaptation, the leaves were exposed to a light pulse with a photosynthetic photon flux density of 1500 µmol m^− 2^ s^− 1^, which allowed the following parameters to be determined: (i) photochemical quenching—qP, (ii) non-photochemical quenching—NPQ, (iii) effective quantum yield of photosystem II—ΔF/Fm’ and (iv) relative electron transport rate—ETR.

#### Biochemical evaluation

Fresh samples of grass leaves were collected in the field and identified according to each treatment. The samples were then frozen in liquid nitrogen and stored in an ultra-low temperature freezer at − 80 °C to preserve the integrity of the plant tissue for later biochemical analysis.

#### Chlorophyll *a*, chlorophyll *b*, total chlorophylls and total carotenoids

A method adapted from Lichtenthaler and Buschmann (2001) was used to determine the levels of chlorophyll *a* (Chl-*a*), chlorophyll *b* (Chl-*b*), total chlorophylls (Total-Chl), and total carotenoids (Total-Carot). Samples of 0.2 g of leaf tissue were weighed and dissolved in 2.5 mL of 80% acetone, then centrifuged at 6000×*g* for 15 min at 4 °C to collect the supernatant. Here, readings were taken using a spectrophotometer (Biochrom, Libra S8, Cambridge, England) at wavelengths of 470, 645 and 663 nm. The levels of chlorophyll *a*, chlorophyll *b*, total chlorophylls and total carotenoids were expressed in mg g^− 1^ of fresh matter (FM) and calculated using the following equations:

$${\mathrm{Chl-}\textit{a}} = \left[ {\frac{{{\mathrm{(12}}{\mathrm{.25}} \times {\textit{A}}_{{{\mathrm{663}}}} - {\mathrm{2}}{\mathrm{.79}} \times {\textit{A}}_{{{\mathrm{645}}}} {\mathrm{)}}}}{{1000 \times W}}} \right] \times {\mathrm{V}} $$

$${\mathrm{Chl-}\textit{b}} = \left[ {\frac{{{\mathrm{(21}}{\mathrm{.5}} \times {\textit{A}}_{{{\mathrm{645}}}} - {\mathrm{5}}{\mathrm{.10}} \times {\textit{A}}_{{{\mathrm{663}}}} {\mathrm{)}}}}{{1000 \times W}}} \right] \times V $$

$$\:\mathrm{Total}\mathrm{-Chl}\text{}\text{ = }{\mathrm{Chl-}\textit{a}} + {\mathrm{Chl-}\textit{b}}$$

$${\text{Total-Carot = }}\left[ {\frac{{\left( {{\mathrm{1000}} \times {\textit{A}}_{{{\mathrm{470}}}} } \right) - \left( {1.82 \times {\mathrm{Chl-}\textit{a}}} \right) - \left( {84.02 \times {\mathrm{Chl-}\textit{b}}} \right)}}{{{\mathrm{198}}}}} \right] $$

where *A* is the absorbance; V is the final extract volume (2.5 mL); W is the weight in grams of the plant tissue (0.2 g).

#### Lipid peroxidation

Lipid peroxidation was determined as per the methodology described by Coelho Júnior et al. (2019), with adaptations based on the quantification of thiobarbituric acid-reactive substances (TBARS). Samples of 0.1 g of leaf tissue were homogenised in 1 mL of 6% trichloroacetic acid (TCA) and then centrifuged at 7960 × *g* for 15 min at 4 °C. After centrifugation, 0.5 mL of the supernatant was collected, to which 1.5 mL of a solution containing 0.5% thiobarbituric acid (TBA) in 20% TCA was added. The mixture was heated to 95 °C for 30 min and then cooled in an ice bath for a further five minutes. Readings were taken using a UV–VIS spectrophotometer (Biochrom, Libra S8, Cambridge, England) at wavelengths of 532 and 660 nm. The TBARS content was calculated using an absorption coefficient of 155 mmol^− 1^ cm^− 1^ and expressed in nmol g^− 1^ FM.

#### Obtaining the enzyme extract

To obtain the enzyme extract, 0.1 g of leaf tissue was weighed, macerated with liquid nitrogen and homogenised with 2 mL of 0.1 M potassium phosphate buffer (pH 7.0) containing 10 mM EDTA, 200 mM ascorbic acid (AsA), and 1% (w/v) insoluble polyvinyl polypyrrolidone (PVPP). The material was then centrifuged at 12,000×*g* for 23 min at 4 °C.

#### Total soluble proteins (TSP), and catalase (CAT), ascorbate peroxidase (APX) and superoxide dismutase (SOD) activity

The total soluble protein content was determined using the methodology adapted from Bradford (1976). A 25 µL sample of the supernatant (obtained as described in section above) was added to 75 µL of ultrapure water and 1000 µL of Bradford’s solution, and then homogenised using a tube shaker. The mixture was allowed to rest for 15 min before readings were taken using a UV–VIS spectrophotometer (Biochrom, Libra S8, Cambridge, England) at 595 nm. The protein concentration was estimated from a standard curve prepared using bovine serum albumin (BSA), and expressed as mg of protein per gram of fresh matter (mg protein g^− 1^ FM).

Catalase activity (CAT) was determined as per the methodology adapted from Cakmak et al. (1993) and Havir and McHale (1987), using 5 µL of enzyme extract (obtained as per item 2.4.6), 45 µL of ultrapure water and 900 µL of 50 mM potassium phosphate buffer (pH 7.0), maintained in a water bath at 27 °C. Before taking the readings, 50 µL of H₂O₂ (20 mM) was added. The readings were taken using a UV-VIS spectrophotometer (Biochrom, Libra S8, Cambridge, England), monitoring the absorbance decay at 240 nm every 10 s for 1.5 min. The catalase activity was calculated based on a molar extinction coefficient of 39.4 mM^− 1^ cm^− 1^ for H₂O₂, and expressed in µmol H₂O₂ mg^− 1^ protein min^− 1^.

Ascorbate peroxidase (APX) activity was determined following the methodology adapted from Nakano and Asada (1981). For the assay, 10 µL of the enzyme extract (obtained as per item above) and 940 µL of 50 mM potassium phosphate buffer (pH 6.0) containing 0.5 mM ascorbic acid were placed in a water bath for five minutes at 27 °C, after which 50 µL of H₂O₂ (30 mM) was added. Readings were taken using a UV-VIS spectrophotometer (Biochrom, Libra S8, Cambridge, England) at 290 nm, recording the absorbance decay every 10 s for 1.5 min. The APX activity was calculated based on a molar extinction coefficient of 2.8 mM^− 1^ cm^− 1^ and expressed in µmol AsA mg^− 1^ protein min^− 1^.

Superoxide dismutase (SOD) activity was determined based on the method described by Giannopolitis and Ries (1977), with adaptations. For the reaction, 10 µL aliquots of the supernatant were added to 40 µL of ultrapure water, 830 µL of potassium phosphate buffer (50 mM; pH 7.8) containing 1 mM EDTA and 13 mM methionine, 100 µL of nitro blue tetrazolium (NBT) (750 µM), and 20 µL of riboflavin (1 mM). After homogenization, the samples were exposed to light in a chamber equipped with 18 W fluorescent lamps for 15 min. Readings were then taken using a spectrophotometer (Biochrom, Libra S8, Cambridge, England) at 560 nm. Enzyme activity was estimated based on the inhibition of NBT reduction, with one unit of activity defined as the amount of enzyme required to inhibit 50% of NBT photoreduction. Results were expressed in U mg^−1^ protein min^−1^.

#### Total soluble carbohydrates, and levels of sodium (Na^+^) and potassium (K^+^)

The extract for determining the sodium (Na^+^), potassium (K^+^) and total soluble carbohydrate content was obtained by weighing 0.2 g of fresh leaf tissue, which underwent extraction by incubation in screw-capped test tubes containing 10 mL of ultrapure water. The tubes were then placed in a water bath at 100 °C for 1 h. The sodium (Na⁺) and potassium (K⁺) content was determined by flame photometry, as described by Rodrigues et al. (2013), with adaptations. After filtering the extracts, the Na⁺ and K⁺ content was analysed using a flame photometer (Micronal, B462, São Paulo, Brazil) based on the standard curves for NaCl and KCl. The results were expressed in µmol g^− 1^ FM.

The total soluble carbohydrate content was determined using the modified methodology of Dubois et al. (1956), for which 25 µL of extract, 475 µL of ultrapure water, 500 µL of 5% phenol and 2500 µL of sulphuric acid (p.a.) were added to test tubes which were stirred by magnetic stirrer and left to rest for 15 min in a water bath at room temperature. The readings were then taken using a spectrophotometer (Biochrom, Libra S8, Cambridge, England) at a wavelength of 490 nm. An anhydrous glucose solution was used to generate the calibration curve. The total soluble carbohydrate content was expressed in g of carbohydrates per 100 g of fresh matter.

#### Leaf area index (LAI), total dry matter (TDM) and harvest index (HI)

Throughout the cycle, biometric evaluations were made regularly every 15 days on two plants from the working area (two central rows) of each plot. The number of live leaves (NLL, units) on each plant was recorded, as well as the length (LL3+, cm) and width (LW3+, cm) of the third fully expanded leaf measured from the apex to the base of the plant using a tape measure. From these variables, the green-leaf area (LA, m^2^) was determined per plant using the methodology proposed by Herman and Câmara (1999):

$${\text{LA = LL}} \times {\mathrm{LW}} \times {\mathrm{0}}{\mathrm{.75}} \times {\text{(NLL + 2)}}$$

where 0.75 is the correction factor for the leaf area of the crop (dimensionless); 2 represents the correction factor for the number of fully open leaves with a green-leaf area of at least 20%, starting from leaf 3+ (dimensionless).

The leaf area index (LAI, m^2^ m^-2^) was determined as the ratio of the leaf area of the plant to the area of soil surface occupied by the plant, as per Watson (1947):

$$\:\mathrm{LAI=}\frac{\mathrm{LA}}{\mathrm{(A}\mathrm{S}\mathrm{OP)}}\:\:\:\:\:\:\:\:\:\:\:\:\:\:\:\:\:\:\:\:\:\:\:\:\:\:\:\:\:\:\:\:$$

where ASOP is the area of soil occupied by the plant (m^2^).

In this study, to determine the total dry matter at the end of the cycle, the number of plants in a two-metre area was counted, considering the two central rows. This procedure allowed the final plant density per hectare to be estimated. Eight plants from this area were then selected, harvested and separated into stems and leaves. Each fraction was placed in kraft paper bags and dried in a forced air circulation oven at 65 °C to constant weight. The values for dry matter were then multiplied by the plant density per hectare to estimate the total production of dry matter, expressed in Mg ha^− 1^.

The harvest index (HI) was determined using the direct cutting method, associated with the morphological separation of plant components (leaves and stems), followed by weighing the fresh biomass and subsequent determination of dry matter. The calculation was performed according to the equation described by Silveira et al. (2026):

$$\:HI=\frac{LDM}{TDM}$$

where LDM corresponds to leaf dry matter (kg) and TDM to total dry matter (kg).

### Statistical analysis

The data were submitted to tests of normality (Shapiro-Wilk) and homoscedasticity (Levene). A two-way analysis of variance (ANOVA) was then carried out, considering the irrigation depths (50%, 75%, 100% and 125% of the ET_0_) and the cycles (first and second cycles) as factors. Whenever the F-test indicated significance and there was an interaction between the factors, the mean values were compared using Tukey’s HSD test (*p* < 0.05). When there was no significant interaction, regression curves were fitted for the factor, irrigation depth, while Tukey’s HSD test was carried out for the factor, cycle (*p* < 0.05). Principal component analysis (PCA) was applied using the mean values of the variables under evaluation, considering eigenvalues greater than 1.0 (Jardim et al. 2025). The data were processed in Microsoft Excel©, while the statistical analysis was carried out using the R software.

## Results

**Fig. 3:**
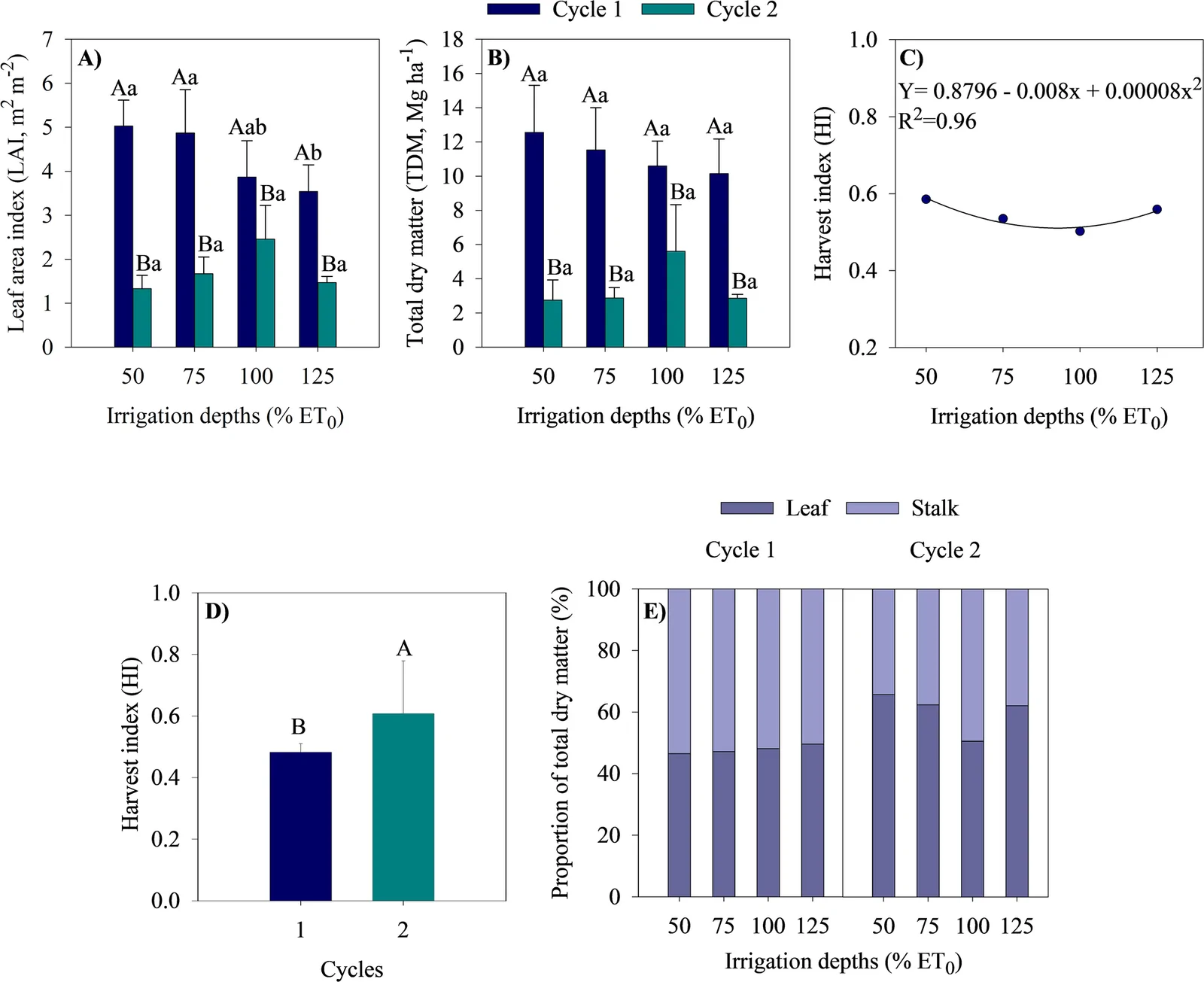
Leaf area index—LAI (**A**), total dry matter—TDM (**B**), harvest index—HI (**C** and **D**), and proportion of total dry matter (**E**) during two cycles of ‘BRS Capiaçu’ grass under different irrigation depths (50%, 75%, 100% and 125% of the reference evapotranspiration—ET_0_). Different letters indicate significant differences by Tukey’s HSD test at 5% probability (*p* < 0.05). Uppercase letters indicate a significant difference between cycles. Lowercase letters indicate a significant difference between irrigation levels within each cycle. The bars at the top of the columns represent the standard deviation of the mean

The leaf area index (LAI) and total dry matter (TDM) showed a significant interaction between irrigation depth and cycle (*p* < 0.05) (Fig. 3A). During Cycle 1, irrigation depths of 50 and 75% of the ET_0_ resulted in higher values for LAI (39.83%), with averages of 5.03 and 4.87 m^2^ m^− 2^, respectively, compared to the irrigation depth of 125%, of 3.54 m^2^ m^− 2^ while during Cycle 2, LAI values were not affected by the irrigation depth (Fig. 3A). TDM did not vary as a function of irrigation during either of the cycles (*p* < 0.05); on the other hand, the highest means were seen in Cycle 1, with averages from 10.15 to 12.56 Mg ha^− 1^, and 2.75 to 5.61 Mg ha^− 1^ for Cycle 2 (Fig. 3B). The harvest index (HI) did not show a significant interaction between the evaluated factors. However, the HI presented higher values during the second cycle, averaging 0.60, compared to 0.48 in the first cycle (Fig. 3D). Regarding irrigation depths, the HI showed quadratic behaviour, with values ranging from 0.50 at 100% ET_0_ to 0.58 at 50% (Fig. 3D). In terms of total dry matter proportion, it can be observed that the stem had a greater share of biomass in Cycle 1, while in Cycle 2, the leaf was the component that exerted the greatest influence on the total biomass (Fig. 3E).

**Fig. 4:**
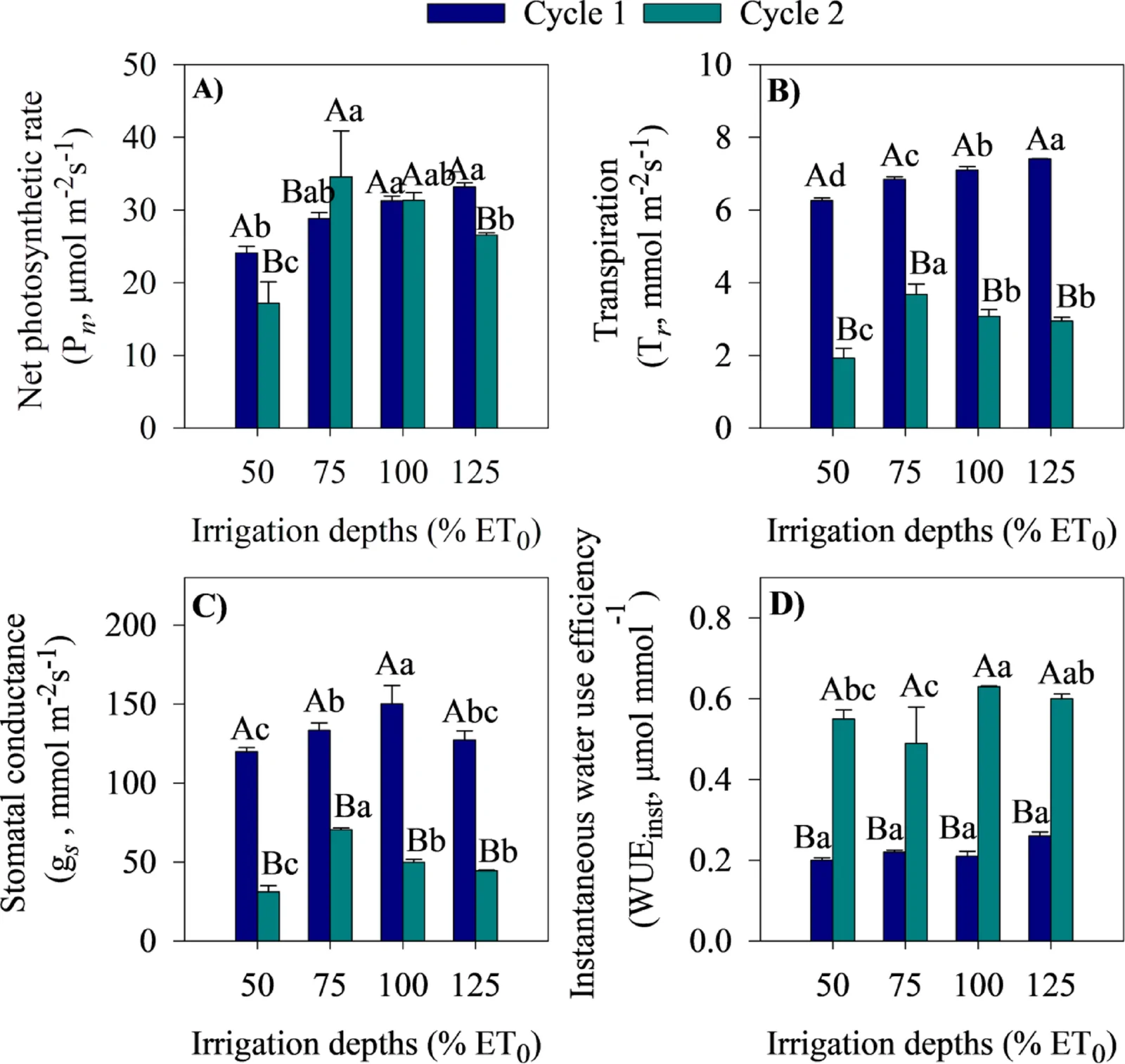
Net photosynthetic rate—P_*n*_ (**A**), transpiration—T_*r*_ (**B**), stomatal conductance—g_*s*_ (**C**) and instantaneous water use efficiency—WUE_inst_ (**D**) during two cycles of ‘BRS Capiaçu’ grass under irrigation depths of 50%, 75%, 100% and 125% of the reference evapotranspiration (ET_0_). Different letters indicate significant differences by Tukey’s HSD test at 5% probability (*p* < 0.05). Uppercase letters indicate a significant difference between cycles. Lowercase letters indicate a significant difference between irrigation levels within each cycle. The bars at the top of the columns represent the standard deviation of the mean

The net photosynthetic rate (P_*n*_), transpiration (T_*r*_), stomatal conductance (g_*s*_) and instantaneous water use efficiency (WUE_inst_) showed significant interactions (*p* < 0.05) between the factors (Fig. 4A). During the second cycle, the highest P_*n*_ values were observed at irrigation depths of 75% and 100% of ET_0_, averaging 32.94 µmol m^− 2^ s^− 1^, a performance 24% better than at the 125% level, which showed 26.55 µmol m^− 2^ s^− 1^. During the first cycle, the irrigation depths of 100% and 125% of the ET_0_ afforded the highest photosynthetic rates, on average 32.21 µmol m^− 2^ s^− 1^, exceeding the 50%, 24.13 µmol m^− 2^ s^− 1^, level by 33%. For T_*r*_ and g_*s*_, the highest values were recorded during Cycle 1, being around three times higher for T_*r*_ and four times higher for g_*s*_ compared to Cycle 2 (Fig. 4B and C). In Cycle 1, transpiration increased proportionally to the irrigation depth, with averages ranging from 6.26 mmol m⁻² s⁻¹ at the 50% ET_0_ depth to 7.41 mmol m⁻² s⁻¹ at the 125% depth (Fig. 4B). In contrast, in Cycle 2, the peak transpiration was observed at the 75% ET_0_ depth, with 3.68 mmol m⁻² s⁻¹ (Fig. 4B). In turn, stomatal conductance showed the highest average value at 100% of ET_0_ during the first cycle, 150.12 mmol m^− 2^ s^− 1^, with an increase of 25.31% compared to the 50% irrigation depth, which showed the lowest g_*s*_, 119.79 mmol m^− 2^ s^− 1^ (Fig. 4C).

In terms of instantaneous water use efficiency (WUE_inst_), the highest values were seen during Cycle 2, particularly at 100% of the ET_0_, when the mean value reached 0.63 µmol mmol^− 1^ — an increase of 15.24% compared to the other depths (Fig. 4D). During Cycle 1, however, there was no significant difference between treatments, with mean values of around 0.26 µmol mmol^− 1^ (Fig. 4D).

**Fig. 5:**
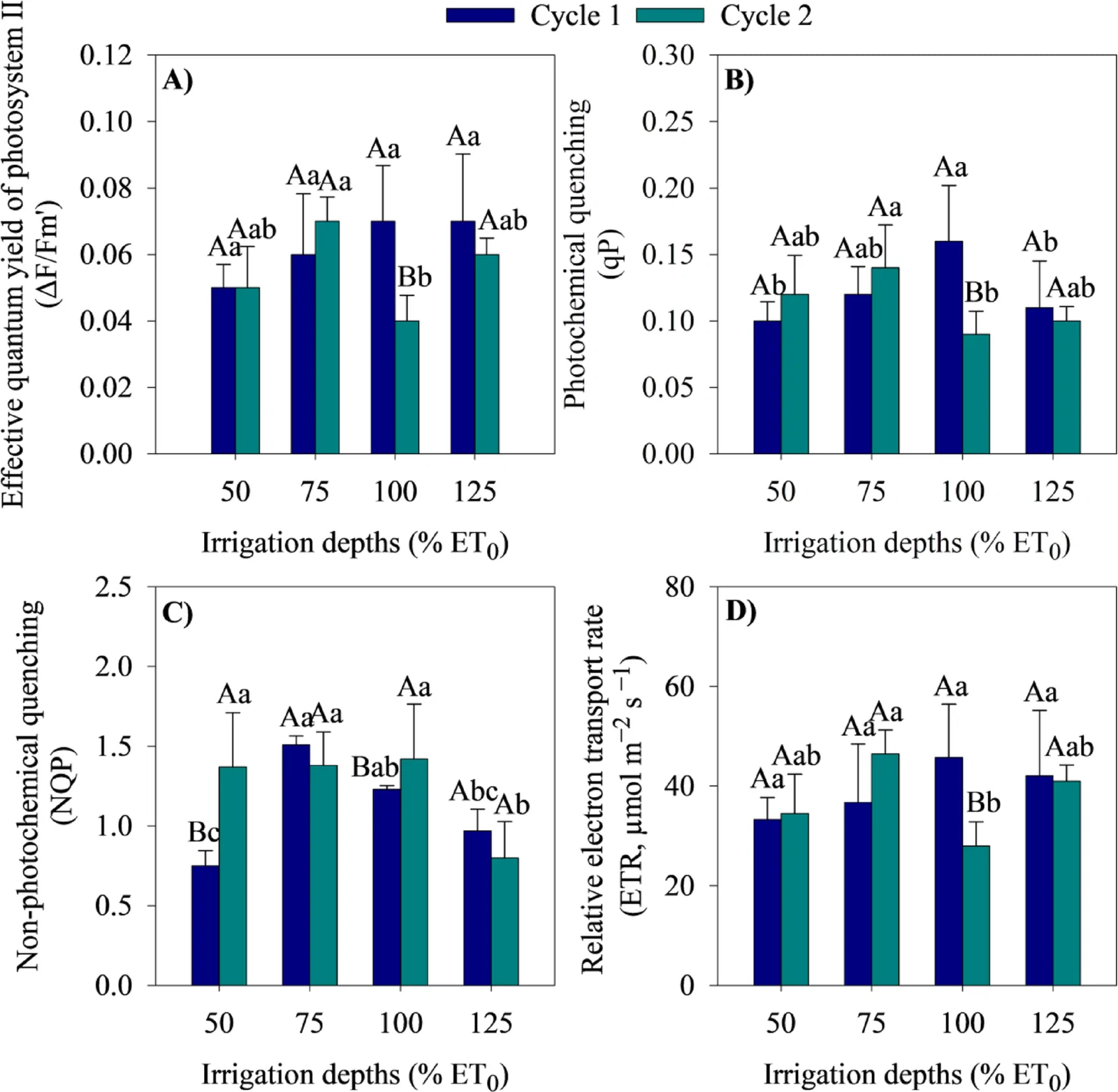
Effective quantum yield of photosystem II—ΔF/Fm’ (**A**), photochemical quenching—qP (**B**), non-photochemical quenching—NQP (**C**) and relative electron transport rate—ETR (**D**) during two cycles of ‘BRS Capiaçu’ grass under different irrigation depths (50%, 75%, 100% and 125% of the reference evapotranspiration—ET_0_). Different letters indicate significant differences by Tukey’s HSD test at 5% probability (*p* < 0.05). Uppercase letters indicate a significant difference between cycles. Lowercase letters indicate a significant difference between irrigation levels within each cycle. The bars at the top of the columns represent the standard deviation of the mean

The effective quantum yield of photosystem II (ΔF/Fm’), photochemical quenching (qP), non-photochemical quenching (NPQ) and relative electron transport rate (ETR) showed significant responses (*p* < 0.05), with interactions between the factors under evaluation (Fig. 5). During Cycle 1, there were no significant differences between the irrigation depths for ΔF/Fm’ or ETR. However, during Cycle 2, the irrigation depth corresponding to 100% of the ET_0_ resulted in the lowest values for these variables compared to the other depths (Fig. 5A and D). For qP, during the first cycle, the highest values were recorded at irrigation depths of 75% and 100% of the ET_0_. During the second cycle, the best performances were observed at irrigation depths of 50% and 75% of ET_0_, with an average of 0.13, a value approximately 44% higher than that obtained at the 100% depth, of 0.09 (Fig. 5B).

For NPQ, the highlight during Cycle 1 was the depth of 75% of the ET_0_, which afforded a 53% increase. During Cycle 2, the depths of 50%, 75% and 100% of the ET_0_ achieved the highest values for non-photochemical quenching, reaching an increase of up to 73% compared to the other depths (Fig. 5C).

**Fig. 6:**
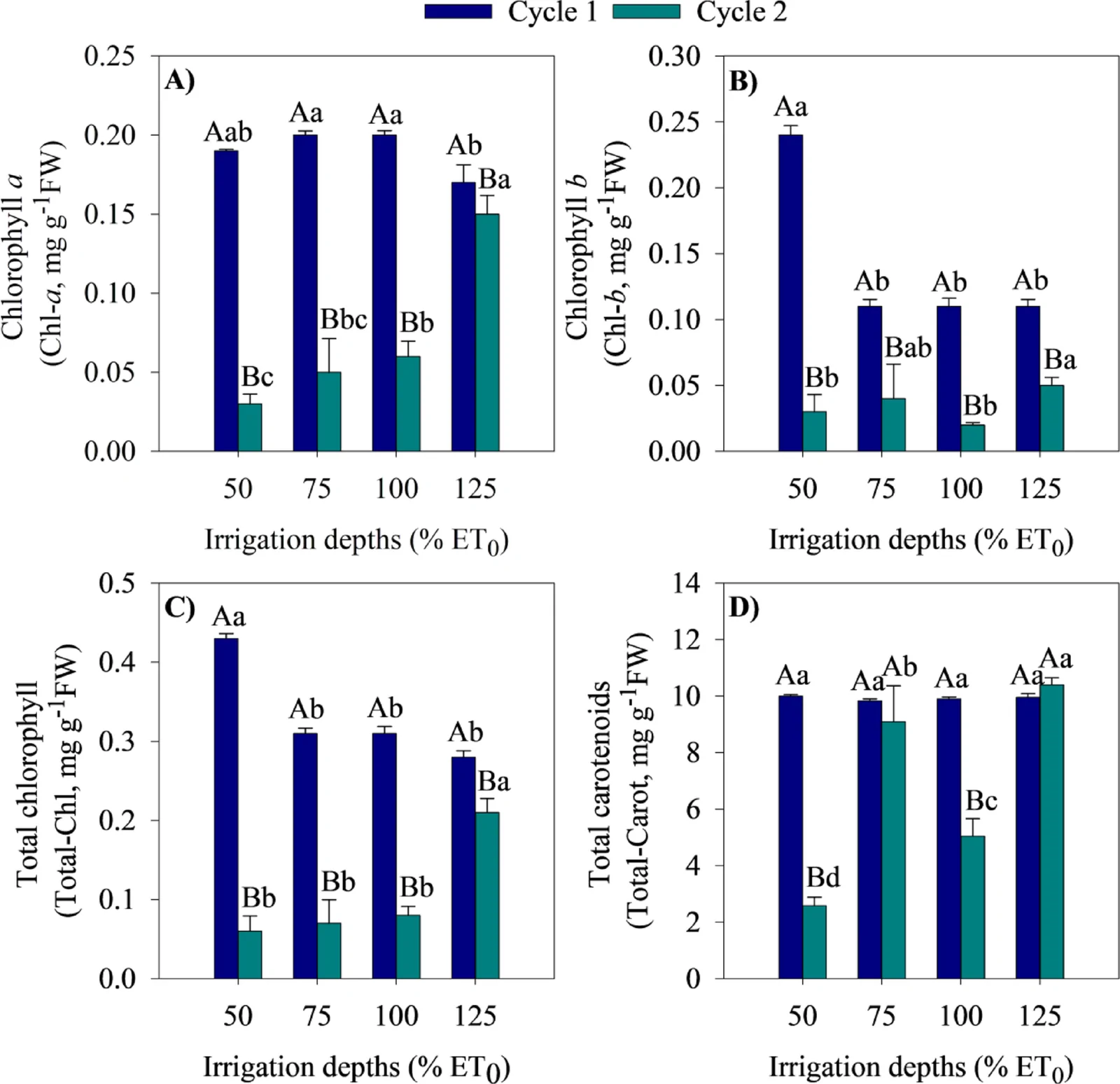
Chlorophyll *a* (**A**), chlorophyll *b* (**B**), total chlorophyll (**C**) and total carotenoids (**D**) during two cycles of ‘BRS Capiaçu’ grass under different irrigation depths (50%, 75%, 100% and 125% of the reference evapotranspiration—ET_0_). Different letters indicate significant differences by Tukey’s HSD test at 5% probability (*p* < 0.05). Uppercase letters indicate a significant difference between cycles. Lowercase letters indicate a significant difference between irrigation levels within each cycle. The bars at the top of the columns represent the standard deviation of the mean

There was a significant interaction (*p* < 0.05) between the factors under evaluation for the levels of chlorophyll *a*, chlorophyll *b* and total chlorophylls, with higher values during the first cycle at each of the irrigation depths (Fig. 6A–C). The chlorophyll *a* content was 11% higher at irrigation depths of 75% and 100% of the ET_0_, while the chlorophyll *b* and total chlorophyll contents were highest at 50% of the ET_0_ (Fig. 6A and B). In terms of the total carotenoid content, there were no significant differences between the irrigation depths during the first cycle. During the second cycle, however, the depths of 75% and 125% of the ET_0_ afforded higher levels of carotenoids compared to the other depths (Fig. 6D).

**Fig. 7:**
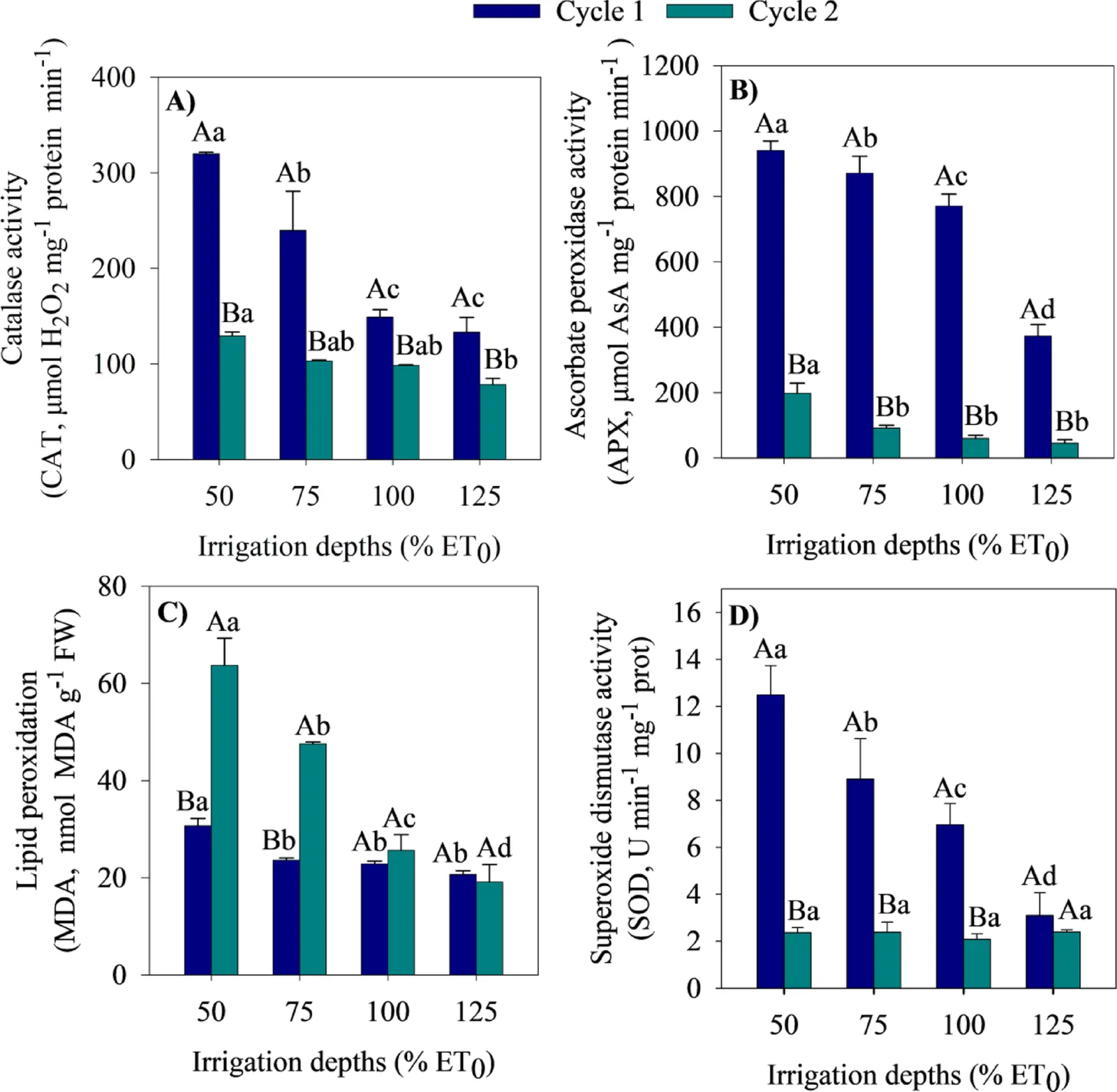
Catalase activity—CAT (**A**), ascorbate peroxidase activity—APX (**B**), lipid peroxidation—MDA (**C**), and superoxide dismutase activity—SOD (**D**) during two cycles of ‘BRS Capiaçu’ grass under irrigation depths of 50%, 75%, 100% and 125% of the reference evapotranspiration (ET_0_). Different letters indicate significant differences by Tukey’s HSD test at 5% probability (*p* < 0.05). Uppercase letters indicate a significant difference between cycles. Lowercase letters indicate a significant difference between irrigation levels within each cycle. The bars at the top of the columns represent the standard deviation of the mean

There was a significant interaction (*p* < 0.05) between the factors under evaluation for catalase (CAT), ascorbate peroxidase (APX), superoxide dismutase (SOD) activity and lipid peroxidation (MDA) (Fig. 7). Higher activity of the antioxidant enzymes catalase (CAT), ascorbate peroxidase (APX), and superoxide dismutase (SOD) was observed during Cycle 1, especially in plants irrigated with 50% of ET_0_ (Fig. 7A, B, and D). Comparing the cycles, Cycle 2 showed a significant reduction in the activity of these enzymes, with values lower than those recorded in Cycle 1. SOD activity ranged from 3.09 to 12.48 U mg⁻¹ of protein min⁻¹ in the first cycle, while in the second cycle the values ranged between 2.07 and 2.39 U mg⁻¹ of protein min⁻¹ (Fig. 7D). In contrast, lipid peroxidation, estimated by malondialdehyde (MDA) content, was higher in Cycle 2, with the highest values observed in plants irrigated with 50 and 75% of ET_0_, reaching 63.68 and 47.55 nmol MDA g^− 1^ FM, respectively (Fig. 7C).

**Fig. 8:**
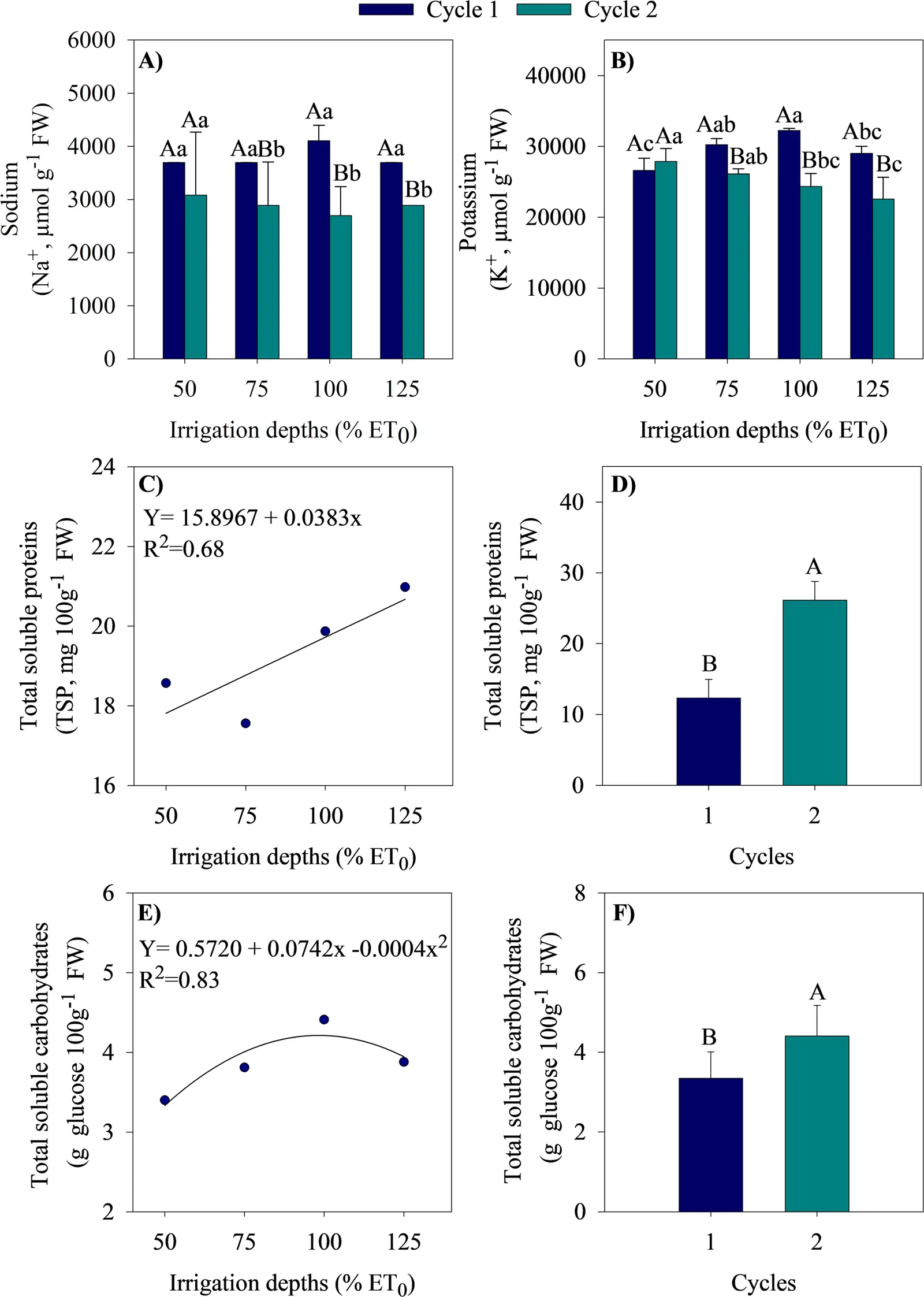
Sodium—Na^+^ (**A**), potassium—K^+^ (**B**), total soluble proteins (**C** and **D**), and total soluble carbohydrates (**E** and **F**) during two cycles of ‘BRS Capiaçu’ grass under different irrigation depths (50%, 75%, 100% and 125% of the reference evapotranspiration—ET_0_). Different letters indicate significant differences by Tukey’s HSD test at 5% probability (*p* < 0.05). Uppercase letters indicate a significant difference between cycles. Lowercase letters indicate a significant difference between irrigation levels within each cycle. The bars at the top of the columns represent the standard deviation of the mean

There was a significant difference (*p* < 0.05) between factors for the levels of sodium (Na⁺) and potassium (K⁺) (Fig. 8A and B). During the first cycle, there were no significant differences in Na⁺ levels for irrigation depth however, during the second cycle, the 50% ET_0_ irrigation depth resulted in the highest Na⁺ concentration, 3,082.50 µmol g⁻¹ FM, with a 14% increase compared to the 100% depth, 2,697.19 µmol g⁻¹ FM (Fig. 8A). Regarding K⁺ content, during Cycle 1, the highest values were recorded at depths of 75% and 100% of ET_0_, while in the second cycle, the highest concentration was observed at 50% of ET_0_, 24,882 µmol g⁻¹ FM, with a 23% increase compared to the 125% depth, 27,882 µmol g⁻¹ FM (Fig. 8B).

The soluble protein and total soluble carbohydrate content showed no significant interaction between the factors under evaluation. However, both compounds presented higher values during the second cycle compared to the first (Fig. 8D and F). The level of soluble proteins showed a linear increase with an increase in irrigation depth, suggesting that their accumulation was a result of the increased availability of brackish water (Fig. 8C). The total soluble carbohydrate content fit a significant quadratic model, peaking at the irrigation depth of 100% of ET_0_, which provided the best results, averaging 4.41 g of glucose 100^− 1^ FM. The irrigation depth with the lowest content was 50% of ET_0_, 3.40 g of glucose 100^− 1^ FM (Fig. 8E).

**Fig. 9:**
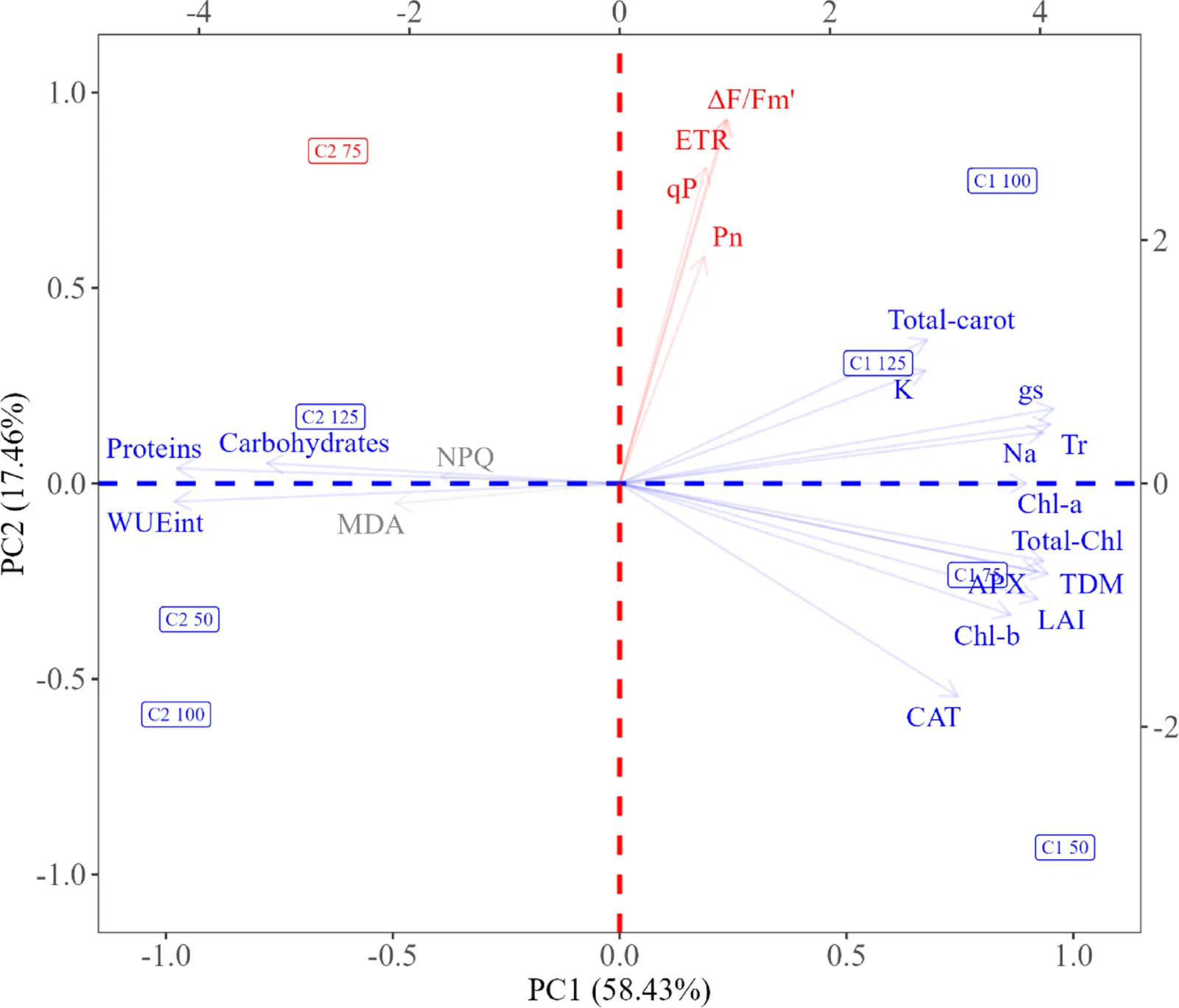
Principal component analysis (PCA) of the productivity variables, photochemical parameters, photosynthetic pigments, and enzyme and non-enzyme antioxidants in the leaves of ‘BRS Capiaçu’ grass over two cycles, under irrigation depths of 50%, 75%, 100% and 125% of the reference evapotranspiration—ET_0_

The principal component analysis explained 75.89% of the total data variance as a function of the irrigation depths of 50%, 75%, 100% and 125% of the ET_0_ applied during two cycles of ‘BRS Capiaçu’, using two principal components (PC1 = 58.43% and PC2 = 17.46%) (Fig. 9). The photosynthetic pigments (Chl-*a*, Chl-*b*, Total-Carot), productivity indices (TDM and LAI), antioxidant enzyme activity (CAT), and physiological parameters (T_*r*_ and g_*s*_) showed positive correlations with each other, and were all positively associated with Cycle 1 and the irrigation depth of 75% of the ET_0_ (Fig. 9). On the other hand, these variables showed a negative correlation with instantaneous water use efficiency (WUE_inst_), as well as with the levels of proteins and carbohydrates, which, in turn, showed a positive correlation with each other and with Cycle 2, especially at 50%, 100% and 125% of the ET_0_ (Fig. 9). In addition, the quantum efficiency of photosystem II (ΔF/Fm’), qP, ETR and the photosynthetic rate (P_*n*_) showed positive correlations with each other and with the depth of 75% of the ET_0_ during Cycle 2 (Fig. 9).

## Discussion

In semi-arid regions, water stress is one of the main challenges to agricultural production, affecting crop development and sustainability (Silva et al. 2021). In livestock farming, this is reflected in the seasonality of forage production, compromising quality and quantity, and animal performance during the dry season (Lara et al. 2021; Maciel et al. 2018). Santos et al. (2025) pointed out that ‘BRS Capiaçu’ shows greater resistance to climate seasonality than do other forage plants, with production ranging from 20 to 30 Mg ha^− 1^. In this study, during Cycles 1 and 2, a productivity of 12 Mg ha^− 1^ was achieved at 75% of the ET_0_ and 5.6 Mg ha^− 1^ at 100% of the ET_0_, respectively (Fig. 3B). The variation in productivity can be explained by the interaction between environmental, physiological, and management factors. During the experiment by Santos et al. (2025), the accumulated rainfall was 2014.4 mm, with an average ET_0_ of 4.59 mm day^− 1^; in contrast, the present study saw an accumulated rainfall of 525.48 mm and an average ET_0_ of 5.11 mm day^− 1^ during the first cycle, and 5.24 mm day^− 1^ during the second, characterising more-severe water stress (Fig. 2). Furthermore, variations in management practices adopted in cultivating ‘BRS Capiaçu’, such as the number regrowth cycles, also influence the results, as the greater this number, the greater the biomass production capacity of the plant (Monção et al. 2019).

Furthermore, during Cycle 2, there was a reduction in the leaf area index (LAI) (Fig. 3A), possibly associated with the high atmospheric demand recorded during this period. The water deficit, calculated as the difference between evapotranspiration and the accumulated rainfall, was 539.6 mm during Cycle 2, while during Cycle 1 it was only 176.61 mm (Fig. 2). The reduction in LAI under severe water stress is related to reduced turgor pressure in the leaves and the reduced availability of photoassimilates. Water scarcity compromises cell rigidity, resulting in a loss of turgor and, consequently, a reduction in the leaf area available for photosynthesis (Taiz et al. 2017).

The harvest index (HI) is a fundamental attribute of productivity, determined by the interaction between genetic, phenological, environmental factors and stress conditions. Although Cycle 1 showed higher dry matter productivity, the harvest index was higher in Cycle 2 (Fig. 3D), indicating that, during this period, the crop directed a greater proportion of photoassimilates to leaf formation, while in Cycle 1, there was a greater allocation of these compounds to other parts of the plant (Singh et al. 2025). This interpretation is corroborated by the dry matter proportion graph (Fig. 3E), which shows that, in Cycle 1, there was a greater accumulation of biomass in the stem compared to the leaves, a behaviour opposite to that observed in Cycle 2, which explains the higher HI in the latter, since this index corresponds to the ratio between leaf dry matter and total dry matter.

The net photosynthetic rate (P_*n*_), which represents the difference between gross photosynthesis and respiration, is essential for plant growth, as it is directly related to biomass accumulation, where higher values indicate increased carbon assimilation and conversion to dry matter (Sudhakar et al. 2016; Keller et al. 2022). In the present study, the best results for P_*n*_ were seen at 75% and 100% of the ET_0_ during Cycle 2 (Fig. 4A), similar results to those reported by Andrade et al. (2024), who identified greater tolerance to water deficit in sugarcane irrigated with 85.16% of the crop evapotranspiration. During Cycle 1, the lower water availability led to a reduction in the photosynthetic rate (Fig. 4A), probably due to partial stomatal closure, a plant strategy to minimise water loss. This behaviour was confirmed by the reduction in transpiration (Fig. 4B) and stomatal conductance (Fig. 4C). Thus, it is evident that lower water availability promoted lower P_*n*_ values, in agreement with the results reported by Dai et al. (2022), when evaluating the effects of supplemental irrigation on wheat cultivation. Stomatal conductance (g_*s*_), a variable that regulates the water vapour flux from the leaf mesophyll to the atmosphere, is highly sensitive to environmental conditions (Lima et al. 2016). In ‘BRS Capiaçu’, the lowest value for g_*s*_ was seen under 50% of the ET_0_ during both cycles (Fig. 4C), a direct reflection of water limitation, which induces stomatal closure as a mechanism for conserving water. This response is common in grasses, and constitutes an essential physiological strategy for survival in water-stressed environments (Silva et al. 2015; Simões et al. 2021), as shown by the reduced volume of water lost through transpiration (Fig. 4B).

Measuring the water use efficiency of plants is essential to understanding the relationship between carbon assimilation and the water lost through transpiration during gas exchange. This knowledge is particularly important in arid and semi-arid regions, where water scarcity requires the rational use of available resources (Cajazeira et al. 2018; Miranda et al. 2023). In the present study, the highest value for instantaneous water use efficiency (WUE_inst_) was recorded during the second cycle (Fig. 4D), precisely the period most affected by the lack of water (Fig. 2). This increase was directly associated with a reduction in transpiration (Fig. 4B), the main pathway for water loss by plants, highlighting an adaptive mechanism for water conservation. According to Yu et al. (2020), under drought conditions, grasses tend to reduce stomatal conductance, which limits transpiration and, consequently, increases WUE_inst_. The results confirm this adaptive response, as shown by the reduction in transpiration (Fig. 4B) and stomatal conductance (Fig. 4C) during the driest cycle. Thus, the limitation of transpiration stands out as a central mechanism for maintaining water efficiency, contributing to the adaptation and survival of plants in water-restricted environments (Hasanuzzaman et al. 2023).

The effective quantum yield of photosystem II (ΔF/Fm’) is an important indicator of the photochemical performance of photosynthesis, as it reflects the fraction of absorbed light energy that is effectively used in electron transport (ETR), a fundamental stage of the photochemical phase (Maia Júnior 2017; Nascimento et al. 2020). In this study, the irrigation depth of 100% of the ET_0_ resulted in lower values for ΔF/Fm’ and ETR during the second cycle (Fig. 5A and D), suggesting less efficient use of the light energy. This reduction may be associated with water stress during this cycle, which caused a reduction in stomatal aperture that was reflected in lower transpiration (Fig. 4B) and stomatal conductance (Fig. 4C). This limitation on CO_2_ diffusion can compromise the continuous flow of electrons from PSII to the reaction centre, thereby reducing the electrons available for photosynthesis (Martins et al. 2024; Zhang and Liu 2018). According to Silva et al. (2024a, b), electron transport in PSII is directly influenced by such factors as stomatal conductance, transpiration and water use efficiency. When compromised, these factors have a negative impact on the photosynthetic rate, as seen under the stress conditions imposed by irrigation at 50% of the ET_0_ during both cycles.

Photochemical quenching (qP) refers to the fraction of photosynthetic electrons effectively transferred after light is absorbed by the pigments in the reaction centre of photosystem II (PSII), showing that the reaction centres are predominantly in an open state. This process is linked to efficient ATP and NADPH consumption in the Calvin-Benson cycle, allowing electrons to be released from the quinone acceptor in PSII and contributing to the photochemical performance of the plant (Jardim et al. 2021). In the present study, ‘BRS Capiaçu’ had higher values for qP during Cycle 1 at 75% and 100% of the ET_0_, and during Cycle 2 at 50% and 75% (Fig. 5B), indicating more-efficient use of light energy under different water conditions. On the other hand, non-photochemical quenching (NPQ) represents the fraction of absorbed energy that is dissipated as heat, a process regulated by the accumulation of protons in the chloroplast lumen and essential for the protection of the photosynthetic apparatus (Maxwell and Johnson 2000; Silva et al. 2006). Elevated NPQ values were seen at 75% of the ET_0_ during Cycle 1 and at 50%, 75% and 100% of the ET_0_ during Cycle 2 (Fig. 5C), suggesting that photoprotective mechanisms were activated. The increase in NPQ may indicate the dissipation of excess light energy that, under stress conditions, is not used for producing NADPH, leading to reduced regeneration of the NADP⁺ electron acceptor. This limitation, associated with the fall in gas exchange (Fig. 4B), can cause energy to accumulate in the chloroplast and favour the formation of reactive oxygen species (ROS), which are potentially damaging to the photosynthetic structures (Murchie and Ruban 2020).

In terrestrial plants, photosynthesis occurs in the chloroplasts, whose membranes harbour photosynthetic pigments such as chlorophylls and carotenoids, essential for capturing and converting light energy into chemical energy (Benavides-Mendoza and Francisco-Francisco 2023). In the present study, levels of photosynthetic pigments were negatively affected by salt stress, particularly during Cycle 2, which showed a more pronounced reduction in chlorophyll *a*, chlorophyll *b* and total chlorophylls compared to Cycle 1 (Fig. 6A, B, and C). This reduction can be attributed to the high salt concentration, intensified by the lower rainfall during the cycle, which compromised the photosynthetic rate and the efficiency of photosystem II (Chen et al. 2024; Krausko et al. 2022). In contrast, there was an increase in carotenoid levels during both cycles (Fig. 6D), especially under moderate salinity, indicating a photoprotective response, since these pigments act as important antioxidants (Silva et al. 2022).

These changes in the photosynthetic pigments are closely related to the accumulation of ROS under salt stress, triggering activation of the antioxidant system of the plant. Enzyme activity, particularly catalase (CAT), ascorbate peroxidase (APX) and superoxide dismutase (SOD), was significantly higher at 50% and 75% of the ET_0_ during Cycle 1 (Fig. 7A and B), reflecting activation of biochemical defences against water and salt stress. These enzymes are crucial for decomposing hydrogen peroxide (H_2_O_2_), thereby minimising oxidative damage to the cellular structures (Huang et al. 2024; Zandi and Schnug 2022). SOD is fundamental in the antioxidant enzyme system, being the first line of defence against ROS; its main function is to convert O_2_^•−^ into H_2_O_2_, which is eliminated by other enzymes (Yang et al. 2021). Some studies report that SOD activity increases under mild or short-term water stress, but decreases under severe or long-term stress (Yang et al. 2021), this behaviour is evidenced in this study in Cycle 2, which showed lower SOD, CAT, and APX activity. Conversely, malondialdehyde (MDA), a marker of lipid peroxidation, showed the direct effect of this redox. In Cycle 1, higher CAT, APX and SOD activity resulted in less MDA accumulation, indicating protection of cell membranes (Fig. 7C). In Cycle 2, reduced antioxidant activity led to increased MDA, evidencing greater oxidative damage (Fig. 7C). Thus, the results demonstrate that the lower water availability in Cycle 2, due to less rainfall interference, compromised the efficiency of the antioxidant system, favouring the accumulation of ROS and membrane peroxidation, establishing a direct relationship with water deficit, as reported by Wang et al. (2026).

Furthermore, during Cycle 2, there was an increase in the accumulation of proteins (Fig. 8D) and soluble carbohydrates (Fig. 8F), compounds that act as osmoprotectants and antioxidants. This adaptive response is related to water scarcity and high salt concentrations, helping to maintain osmotic balance and protecting the cells from the harmful effects of stress (Al-Janabia et al. 2024).

Potassium (K⁺) levels showed no significant variation between the different irrigation depths within each cycle (Fig. 8B). This nutrient plays an essential role in several regulatory functions of plant metabolism, including water absorption, osmotic control, and stomatal opening and closing (Chakraborty et al. 2016; Jardim et al. 2021). Similarly, there were no major differences in sodium (Na⁺) levels between the treatments (Fig. 8A). Under water stress, an increase in the Na⁺ concentration in plant tissue is common, since this ion is absorbed more easily than K⁺ (Yadav et al. 2006). According to Ghafar et al. (2021), Na⁺ and K⁺ compete with each other for root absorption; however, in this study, the increase in K⁺ absorption did not result in a reduction in Na⁺ absorption, suggesting an efficient ionic balance even under stress.

The principal component analysis (PCA) was essential for synthesising and interpreting the complex data obtained under water and salt stress (Fig. 9). The results showed that Cycle 1, when combined with irrigation at 75% of the ET_0_, was associated with more-favourable conditions, reflected in increased maintenance of photosynthetic pigments and higher rates of productivity, suggesting a lower level of environmental stress (Fig. 9). On the other hand, during Cycle 2, characterised by more-severe stress, the irrigation depth of 75% of the ET_0_ continued to stand out, demonstrating the ability of ‘BRS Capiaçu’ to maintain photosynthetic rates and physiological efficiency even under adverse conditions, highlighting its adaptability and tolerance to stress (Fig. 9).

This study demonstrated that ‘BRS Capiaçu’ exhibits physiological, biochemical and photosynthetic responses that contribute to its resilience to water and salt stress, which vary depending on the irrigation depth, where intermediate values favoured photosynthesis, stomatal conductance, water use efficiency and productivity, while more-severe conditions resulted in a reduction in photosynthetic pigments and the activation of defence mechanisms. Notable among these were increased levels of carotenoids, proteins and soluble carbohydrates, as well as increased activity of the antioxidant enzymes CAT and APX, which act to neutralise reactive oxygen species. These results underline the importance of proper water management to minimise the negative effects of stress and ensure the sustainability of forage production in semi-arid regions using brackish water.

## Conclusion

The present study demonstrated that salt stress intensifies during periods of less rainfall, making water availability a determining factor in the efficiency of the physiological and photochemical processes of the plants. Irrigation at 50% of the ET_0_ significantly compromised gas exchange parameters and chlorophyll fluorescence, in addition to inducing greater antioxidant enzyme activity, showing that the crop was under physiological stress. In contrast, ‘BRS Capiaçu’ showed excellent physiological and productive performance under a water deficit of 75% and 100% of the ET_0_. Its high tolerance to semi-arid conditions was demonstrated by the efficient modulation of its physiological and biochemical responses, resulting in greater water use efficiency. This adaptive adjustment reinforces the potential of ‘BRS Capiaçu’ as a strategic option for mitigating the effects of seasonality on forage production in regions with a dry climate. It can therefore be concluded that ‘BRS Capiaçu’ grass has a remarkable capacity to adapt to the environmental stresses of the semi-arid region, consolidating its position as a promising solution for the sustainability of agricultural production in these areas.

## Data Availability

The original contributions presented in this study are included in the article. Further inquiries can be directed to the corresponding authors.

## References

[CR1] Al-Janabia AMI, Al-Dulaimy AFZ, Sekhi YS, Almohammedi OHM, Al-Taey DKA (2024) Effect of salt stress on growth and yield of plants: a review. IOP Conf Series: Earth Environ Sci 1371(4):042028. 10.1088/1755-1315/1371/4/042028

[CR2] Allen RG (1998) Crop evapotranspiration: guidelines for computing crop water requirements. F.A.O

[CR3] Alvares CA, Stape JL, Sentelhas PC, de Moraes Gonçalves JL, Sparovek G (2013) Köppen’s climate classification map for Brazil. Meteorol Z 22(6):711–728. 10.1127/0941-2948/2013/0507

[CR4] Alves CP, Jardim AM, da Araújo RF, Júnior G, do, Souza N, de Araújo LSB, de Souza GGL, de Salvador CAA, Leite KR, Pinheiro RMC, Silva AG (2022) How to enhance the agronomic performance of cactus-sorghum intercropped system: planting configurations, density and orientation. Ind Crop Prod 184:115059. 10.1016/j.indcrop.2022.115059

[CR5] Andrade WJM, De, Silva GF, Da, Menezes SM, De, Silva MM, Da, Silva AO, Da, Lopes IAP, Filho O, De RA, Sousa RR, De (2024) Características fisiológicas e rendimento da cana-de-açúcar sob lâminas de irrigação. Observatório De La Economía Latinoam 22(1):4387–4415. 10.55905/oelv22n1-232.

[CR6] Araújo Júnior GdoN, Leite RMC, de Morais JEF, Alves CP, de Souza CAA, dos Almeida AC S., Jardim AM, da Souza RF, de Eugenio LSB (2024) Growth dynamic, productivity, evapotranspiration, and water-economic indices of forage cactus under different irrigation depths. Agronomy 14(4):691 10.3390/agronomy14040691

[CR7] Araújo Júnior GDN, Morais JEF, De Souza LSB, de Steidle Neto AJ, Araujo GGL, de Silva TGF (2023) Use of lower quality water in irrigated agriculture and effects on forages with productive potential in semiarid regions: a review. Environ Process 10(3):1–28. 10.1007/S40710-023-00655-6

[CR8] Araújo SADC, Vasquez HM, Campostrini E, Netto AT, Deminicis BB, Da Silva Lima É (2010) Características fotossintéticas de genótipos de capim-elefante anão (*Pennisetum purpureum* Schum.), em estresse hídrico. Acta Scientiarum - Anim Sci 32(1):1–7. 10.4025/actascianimsci.v32i1.8961

[CR9] Benavides-Mendoza A, Francisco-Francisco N (2023) Recientes aplicaciones de la fluorescencia de la clorofila en los cultivos vegetales. Epistemus 10.36790/epistemus.v16i33.285

[CR10] Bradford MM (1976) A rapid and sensitive method for the quantitation of microgram quantities of protein utilizing the principle of protein-dye binding. Anal Biochem 72(1–2):248–254. 10.1016/0003-2697(76)90527-3 942051

[CR11] Cakmak I, Strbac D, Marschner H (1993) Activities of hydrogen peroxide-scavenging enzymes in germinating wheat seeds. J Exp Bot 44(1):127–132. 10.1093/jxb/44.1.127

[CR12] Cajazeira JP, Corrêa MC, de Almeida M, Queiroz EIB, Mesquita RF, R. O (2018) Growth and gas exchange in white pitaya under different concentrations of potassium and calcium. Revista Ciência Agronômica. 10.5935/1806-6690.20180013

[CR13] Cavalcante ES, de Lacerda CF, Costa RNT, Gheyi HR, Pinho LL, Bezerra FMS, de Oliveira AC, Canjá JF (2021) Supplemental irrigation using brackish water on maize in tropical semi-arid regions of Brazil: yield and economic analysis. Scientia Agricola 78:e20200151. 10.1590/1678-992X-2020-0151

[CR14] Cavalcanti FJ, de Santos A, dos JCP, Pereira JR, Leite JP, Silva MCL, da, Freire FJ, da Silva DJ, de Sousa AR, Messias AS, de Faria CMB, Junior BNL, Gomes MA, Cavalcanti RV (2008) A.C., Lima J.F.W.F. Recomendações de adubação para o Estado de Pernambuco: 2^a^ aproximação. Instituto Agronomico de Pernambuco - IPA 83–103

[CR15] Chakraborty K, Bhaduri D, Meena HN, Kalariya K (2016) External potassium (K+) application improves salinity tolerance by promoting Na+-exclusion, K+-accumulation and osmotic adjustment in contrasting peanut cultivars. Plant Physiol Biochem 103:143–153. 10.1016/j.plaphy.2016.02.039 26994338

[CR16] Chen X, Jiang Y, Cong Y, Liu X, Yang Q, Xing J, Liu H (2024) Ascorbic acid mitigates salt stress in tomato seedlings by enhancing chlorophyll synthesis pathways. 14(8):1810. 10.3390/AGRONOMY14081810

[CR17] Chilundo M, Joel A, Wesström I, Brito R, Messing I (2016) Effects of reduced irrigation dose and slow release fertiliser on nitrogen use efficiency and crop yield in a semi-arid loamy sand. Agric Water Manage 168:68–77. 10.1016/j.agwat.2016.02.004

[CR18] Coelho Júnior LF, Ferreira-Silva SL, Vieira MR, Carnelossi MA, Simoes AN (2019) Darkening, damage and oxidative protection are stimulated in tissues closer to the yam cut, attenuated or not by the environment. J Sci Food Agric 99(1):334–342. 10.1002/jsfa.9192 29885065

[CR19] Dai Y, Fan J, Liao Z, Zhang C, Yu J, Feng H, Zhang F, Li Z (2022) Supplemental irrigation and modified plant density improved photosynthesis, grain yield and water productivity of winter wheat under ridge-furrow mulching. Agric Water Manag 274:107985. 10.1016/J.AGWAT.2022.107985

[CR62] da Silva MMP, Vasquez HM, Bressan-Smith R, da Silva JFC, Erbesdobler ED, de Andrade Junior PSC (2006) Eficiência fotoquímica de gramíneas forrageiras tropicais submetidas à deficiência hídrica. Revista Brasileira de Zootecnia 35(1):67–74. 10.1590/S1516-35982006000100008

[CR77] da Silva MV, Pandorfi H, Jardim AM, da Oliveira-Júnior RF, de Divincula JF, da, Giongo JS, Silva PR, de Almeida TGF, Moura GLP, de Lopes GB (2021) A P. M. O. Spatial modeling of rainfall patterns and groundwater on the coast of northeastern Brazil. Urban Clim 38, 100911. 10.1016/J.UCLIM.2021.100911

[CR58] da Silva AAR, de Lima GS, de Azevedo CAV, Gheyi HR, Soares LADA, de Veloso LL S. A (2022) Salicylic acid improves physiological indicators of soursop irrigated with saline water. Revista Brasileira de Engenharia Agrícola e Ambiental 26(6):412–419. 10.1590/1807-1929/AGRIAMBI.V26N6P412-419

[CR60] da Silva GIN, de Morais JEF, de Souza CAA, Jardim AMDRF, Alves CP, da Silva Salvador KR, da Silva TGF (2024) Do different densities and planting orientations of forage cactus alter agronomic, morphophysiological characteristics, and soil water dynamics in a semiarid region? Eur J Agron 159:127271. 10.1016/J.EJA.2024.127271

[CR78] dos Santos ARM, de Morais JEF, Salvador KR da S., Souza CAA de, Araújo GdoN Júnior, Alves CP, Jardim AM da R. F., Silva MJ da, Carvalho FG de, dos Santos ASL, Santos WR dos, Campos FS, Gois GC, Silva TGF da (2025) Environmental seasonality affects the growth, yield and economic viability of irrigated forage species in dry regions. Irrig Sci. 10.1002/ird.3077

[CR20] Dubois M et al (1956) Colorimetric method for determination of sugars and related substances. Anal Chem 28:350–356

[CR21] Ghafar MA, Akram NA, Saleem MH, Wang J, Wijaya L, Alyemeni MN (2021) Ecotypic morphological and physio-biochemical responses of two differentially adapted forage grasses, Cenchrus ciliaris L. and Cyperus arenarius Retz. Drought Stress Sustain 13(14):8069. 10.3390/su13148069

[CR22] Ghannoum O (2009) C4 photosynthesis and water stress. Ann Bot 103(4):635–644. 10.1093/aob/mcn093 18552367 PMC2707343

[CR23] Giannopolitis CN, Ries SK (1977) Superoxide dismutasesI. Occurrence in higher plants. Plant Physiol 59(2):309–314 16659839 10.1104/pp.59.2.309 PMC542387

[CR24] Habermann E, Dias de Oliveira EA, Contin DR, Delvecchio G, Viciedo DO, de Moraes MA, de Mello Prado R, de Pinho Costa KA, Braga MR, Martinez CA (2019) Warming and water deficit impact leaf photosynthesis and decrease forage quality and digestibility of a C4 tropical grass. Physiol Plant 165(2):383–402. 10.1111/PPL.12891 30525220

[CR25] Hasanuzzaman M, Zhou M, Shabala S (2023) How does stomatal density and residual transpiration contribute to osmotic stress tolerance? Plants 12:494. 10.3390/PLANTS12030494 PMC9919688 36771579

[CR26] Havir EA, McHale NA (1987) Biochemical and developmental characterization of multiple forms of catalase in tobacco leaves. Plant Physiol 84(2):450–455. 10.1104/pp.84.2.450 16665461 PMC1056601

[CR27] Hermann E, Rodrigues (1999) Gil Miguel de Sousa Câmara. Um método para estimar a área foliar da cana-de-açúcar. STAB-Açúcar e Álcool. e Subprodutos 17(5):32–34

[CR28] Huang Y, Li J, Nong C, Lin T, Fang L, Feng X, Chen Y, Lin Y, Lai Z, Miao L (2024) Piriformospora indica enhances resistance to fusarium wilt in strawberry by increasing the activity of superoxide dismutase, peroxidase, and catalase, while reducing the content of malondialdehyde in the roots. Horticulturae 10(3):240. 10.3390/horticulturae10030240

[CR29] Jardim AM, da Souza RF, Alves LSB, Ferreira Nunes de Araújo CP, de Souza J, Pinheiro CAA, de Araújo AG, Campos GGL, Tabosa FS, Silva JN (2023) Intercropping forage cactus with sorghum affects the morphophysiology and phenology of forage cactus. Afr J Range Forage Sci 40(2):129–140 10.2989/10220119.2021.1949749

[CR30] Jardim AM, da Morais RF, de Tang JEF, Souza X, de Souza LSB, de Santos CAA, dos, Marin WR, Alves FR, Silva N, da Leite CP, Salvador RMC, Lopes KR, de Neto S, Ometto D, de Lima AJ, Silva JPHB (2025) JLMP Energy balance, water use efficiency, and photochemistry of two globally cultivated rainfed cactus species. Agric Water Manag 311, 109385. 10.1016/J.AGWAT.2025.109385

[CR31] Jardim AM, da R. F., Santos HRB, Alves HKMN, Ferreira-Silva SL, Souza LSBde, Araújo GdoN Júnior, Souza MdeS, Araújo GGLde, Souza CAAde, Silva TGFda (2021) Genotypic differences relative photochemical activity, inorganic and organic solutes and yield performance in clones of the forage cactus under semi-arid environment. Plant Physiol Biochem 162:421–430. 10.1016/J.PLAPHY.2021.03.011 33740681

[CR32] Karolina A, De P, Silva M, Braga SE, De Holanda M, Rocha R, Vasconcelos LG, Cavalcanti K, M (2025) Convivência com o semiárido: políticas públicas e tecnologias para mitigar a escassez hídrica. Caderno Pedagógico 22(7):e16181–e16181. 10.54033/CADPEDV22N7-094

[CR33] Keller B, Zimmermann L, Rascher U, Matsubara S, Steier A, Muller O (2022) Toward predicting photosynthetic efficiency and biomass gain in crop genotypes over a field season. Plant Physiol 188(1):301–317. 10.1093/PLPHYS/KIAB483 34662428 PMC8774793

[CR34] Krausko M, Kusá Z, Peterková D, Labajová M, Kumar A, Pavlovič A, Bačovčinová M, Bačkor M, Jásik J (2022) The absence of the AtSYT1 function elevates the adverse effect of salt stress on photosynthesis in arabidopsis. Int J Mol Sci 2022 23(3):1751. 10.3390/IJMS23031751 PMC8836111 35163669

[CR35] Kresović B, Tapanarova A, Tomić Z, Životić L, Vujović D, Sredojević Z, Gajić B (2016) Grain yield and water use efficiency of maize as influenced by different irrigation regimes through sprinkler irrigation under temperate climate. Agric Water Manage 169:34–43. 10.1016/j.agwat.2016.01.023

[CR36] Lankford B, Pringle C, McCosh J, Shabalala M, Hess T, Knox JW (2023) Irrigation area, efficiency and water storage mediate the drought resilience of irrigated agriculture in a semi-arid catchment. Sci Total Environ 859:160263. 10.1016/J.SCITOTENV.2022.160263 36402330

[CR37] Lara MAS, Silva VJ, Sollenberger LE, Pedreira CGS (2021) Seasonal herbage accumulation and canopy characteristics of novel and standard brachiariagrasses under N fertilization and irrigation in southeastern Brazil. Crop Sci 61(2):1468–1477. 10.1002/CSC2.20353

[CR38] Lichtenthaler HK, Buschmann C (2001) Chlorophylls and carotenoids: measurement and characterization by spectroscopy. Curr Protocols Food Anal Chem. 10.1002/0471142913.faf0403s01

[CR39] Lima MJ, Farias VD, Costa DL, Sampaio LS, Souza PJ (2016) Efeito combinado das variáveis meteorológicas sobre a condutância estomática do feijão-caupi. Hortic Brasileira 34(4):547–553. 10.1590/s0102-053620160414

[CR40] Maciel GA, Braga GJ, Guimarães R, Ramos AKB, Carvalho MA, Fernandes FD, Fonseca CEL, Jank L (2018) Seasonal liveweight gain of beef cattle on Guineagrass pastures in the Brazilian Cerrados. Agron J 110(2):480–487. 10.2134/AGRONJ2017.05.0262

[CR41] Maia Júnior SDO (2017) Tolerância de cultivares de cana-de-açúcar ao déficit hídrico: relações hídricas, trocas gasosas, fluorescência da clorofila e metabolismo antioxidante. Universidade Federal De Alagoas

[CR42] Martins LDC, de Martim S, Guerra MBT, Brito TM, de Mello FAL, de Júnior NRC, dos Santos WM, Soares AL, do Nascimento JF, da Silva TGF, Santos HRB, Ferreira-Silva SL, Simões A (2024) do N. Physiological and anatomical mechanisms induced by water deficit on the longevity and post-harvest quality of amaryllis stems. Sci Hort, 330, 113082. 10.1016/J.SCIENTA.2024.113082

[CR43] Maxwell K, Johnson GN (2000) Chlorophyll fluorescence—a practical guide. J Exp Bot 51(345):659–668. 10.1093/jexbot/51.345.659 10938857

[CR44] Miranda JR, Furtado DA, Silva VC, da, Dantas Neto J, Souza JTA, Araújo JS (2023) Physiology of the forage cactus cultivate *Opuntia stricta* (Haw.) Haw under different irrigation frequencies in the Semiarid. Revista Ceres 70(1):24–31. 10.1590/0034-737x202370010003

[CR45] Monção FP, Costa MAMS, Rigueria JPS, Moura MMA, Rocha Júnior VR, Gomes VM, Leal DB, Maranhão CMA, Albuquerque CJB, Chamone JMA (2019) Yield and nutritional value of BRS Capiaçu grass at different regrowth ages. Semina: Ciências Agrárias, 40(5), 2045. 10.5433/1679-0359.2019v40n5p2045

[CR46] Moura MMA, Alves WS, Rigueira JPS, Mizobutsi EH, Fernandes MB, de Almeida LB, Araujo IVS (2024) Estrategias de manejo para a cultivar BRS capiaçu: uma revisão de literatura. Brazilian J Anim Environ Res 7(2):e69353. 10.34188/bjaerv7n2-052

[CR47] Murchie EH, Ruban AV (2020) Dynamic non-photochemical quenching in plants: from molecular mechanism to productivity. Plant J 101(4):885–896. 10.1111/TPJ.14601;REQUESTEDJOURNAL:JOURNAL:1365313X;WGROUP:STRING:PUBLICATION 31686424

[CR48] Nakano Y, Asada K (1981) Hydrogen peroxide is scavenged by ascorbate-specific peroxidase in Spinach chloroplasts. Plant Cell Physiol. 10.1093/oxfordjournals.pcp.a076232

[CR49] Nascimento JWS, do Oliveira NPde, Oliveira GCB, Madalena NdaS Júnior, Serafim EdeO, Pereira LS, Bernardo FdaG, Nascimento HH do (2020) Ecofisiologia de mudas de bauhinia forficata link submetidas à supressão de rega e posterior reirrigação/ecophysiology of bauhinia forficata link seedlings submitted to irrigation suppression and subsequent re-irrigation. Brazilian J Dev 6(9):72360–72377. 10.34117/bjdv6n9-617

[CR50] Pereira PDC, Silva TGF, Da, Zolnier S, Morais JEF, De, Santos DC, Dos (2015) Morfogênese da palma forrageira irrigada por gotejamento. Revista Caatinga 28(3):184–195. 10.1590/1983-21252015v28n321rc

[CR51] Pereira A, Vander, Ledo FJdaS, Morenz MJF, Leite JLB, Santos AMB, Dos, Martins CE, Machado JC (2016) BRS Capiaçu: cultivar de capim-elefante de alto rendimento para produção de silagem. In Embrapa Gado de Leite.

[CR52] Prudente Junior AC (2019) Utilização de sonda capacitiva FDR para estimativa do consumo de água e coeficiente de cultura de *Urochloa brizantha* cv *Marandu* em cultivo solteiro e consorciado [Dissertação de Mestrado, Escola Superior de Agricultura Luiz de Queiroz]. 10.11606/D.11.2019.tde-29082019-170608

[CR53] Retore M, Pereira J, Marco A, Orrico AP, Galeano J, J. E (2021) Manejo do capim BRS Capiaçu para aliar produtividade à qualidade. Embrapa

[CR54] Richards LA (1954) Diagnosis and Improvement of Saline and Alkali Soils

[CR55] Rodrigues CRF, Silva EN, Ferreira-Silva SL, Voigt EL, Viégas RA, Silveira JAG (2013) High K+ supply avoids Na+ toxicity and improves photosynthesis by allowing favorable K+: Na+ ratios through the inhibition of Na+ uptake and transport to the shoots of Jatropha curcas plants. J Plant Nutr Soil Sci 176(2):157–164. 10.1002/jpln.201200230

[CR56] Santos HG, Dos, Jacomine PKT, Anjos LHC, Dos, Oliveira VA, De, Lumbreras JF, Coelho MR, Almeida JA, De, Araujo Filho JC, De, Oliveira JB, De (2018) Cunha, T. J. F. Sistema brasileiro de classificação de solos.

[CR57] Santos ARM, dos CA, Bezerra R, Raissa Barros Alves Cordeiro L, Luiz de Mello Vieira Leite M, Renan da Silva Salvador K, Daiane Costa de Sousa, Lady, Carlos, Nogueira J, dos Santos Gomes Calaça, Gomes J, de Carvalho F, Roberta dos Santos, W., George Freire da Silva, Associado T (2023) P. Valor nutritivo de plantas forrageiras cultivadas no semiárido brasileiro. Revista Brasileira de Geografia Física, 16(03), 1466–1489

[CR59] Silva MDA, Lonfover Jifon J, Moura C, Santos D, Jadoski CJ, Da Gonçalves JA (2013) Photosynthetic capacity and water use efficiency in sugarcane genotypes subject to water deficit during early growth phase. Brazi Arch Biol Technol 56(5):735–748. 10.1590/S1516-89132013000500004

[CR61] Silva MSNE, Oliveira VAV, De, Donato LMS, De Souza RF, Duarte SM, Ferreira GA, de Barros P, Santos RE, L. D. T (2024b) Restrição luminosa e o manejo químico com glyphosate sob a resposta fisiológica de *Urochloa brizantha*. Contribuciones las ciencias sociales 17(1):6600–6619. 10.55905/revconv.17n.1-397

[CR63] Silva FG, da, Dutra WF, Dutra AF, de Oliveira IM, Filgueiras LMB, de Melo AS (2015) Trocas gasosas e fluorescência da clorofila em plantas de berinjela sob lâminas de irrigação. Revista Brasileira de Engenharia Agrícola e Ambiental 19(10):946–952. 10.1590/1807-1929/agriambi.v19n10p946-952

[CR64] Silveira DC, Mills A, Cruz CD, Antoniolli J, Bonotto CZ, de Ávila VS, Weiler RL, Brunes AP, Simioni C, Dall’Agnol M (2026) Repeatability, Genetic Variability and Importance of Forage Traits in Ecotypes of Brunswickgrass (Paspalum nicorae Parodi) via BLUP. Plant Breed 0:1–14. 10.1111/PBR.70060;WGROUP:STRING:PUBLICATION

[CR65] Simões WL, Andrade VP, Mouco MA, do C, de Sousa JSC, de Lima JRF (2021) Produção e qualidade da mangueira ‘Kent’ (*Mangifera indica* L.) submetida a diferentes lâminas de irrigação no semiárido nordestino. Revista Em Agronegócio e Meio Ambiente 14(2):e7832. 10.17765/2176-9168.2021v14n2e7832

[CR66] Singh GM, Bhati PK, Vishwakarma MK, Ladejobi F, Mishra VK, Sharma S, Joshi AK (2025) Genome wide dissection and haplotype analysis to identify candidate loci for harvest index under spot blotch in bread wheat. Mol Genet Genom 2025 301(1):5. 10.1007/S00438-025-02306-X 41381936

[CR67] Singh PK, Chudasama H (2021) Pathways for climate change adaptations in arid and semi-arid regions. J Clean Prod 284:124744. 10.1016/j.jclepro.2020.124744

[CR68] Sudhakar P, Latha P, Reddy PV, Traits (2016) 27–31. 10.1016/B978-0-12-804073-7.00003-X

[CR69] Taiz L, Zeiger E, Møller IM, Murphy A (2017) Fisiologia e Desenvolvimento Vegetal (6th ed.)

[CR70] Wang J, Zhang S, Ni S, Zhang J, Qin Y, Wang J, Yang Q (2026) Morpho-anatomical changes and physio-biochemical responses of Carex siderosticta under water stress. Plant Signal Behav. 10.1080/15592324.2026.2658186 42015474 PMC13108358

[CR71] Watson DJ (1947) Comparative physiological studies on the growth of field crops: I. Variation in net assimilation rate and leaf area between species and varieties, and within and between years. Ann Bot 11(41):41–76

[CR72] Yadav SS, Kumar J, Yadav SK, Singh S, Yadav VS, Turner NC, Redden R (2006) Evaluation of Helicoverpa and drought resistance in desi and kabuli chickpea. Plant Genetic Resour 4(3):198–203. 10.1079/PGR2006123

[CR73] Yang X, Lu M, Wang Y, Wang Y, Liu Z, Chen S (2021) Response mechanism of plants to drought stress. Horticulturae 7:50. 10.3390/HORTICULTURAE7030050

[CR74] Yu L, Gao X, Zhao X (2020) Global synthesis of the impact of droughts on crops’ water-use efficiency (WUE): Towards both high WUE and productivity. Agric Syst 177:102723. 10.1016/J.AGSY.2019.102723

[CR75] Zandi P, Schnug E (2022) Reactive oxygen species, antioxidant responses and implications from a microbial modulation perspective. Biology 2022 11(2):155. 10.3390/BIOLOGY11020155 PMC8869449 35205022

[CR76] Zhang Y, Liu G (2018) Effects of cesium accumulation on chlorophyll content and fluorescence of Brassica juncea L. J Environ Radioact 195:26–32. 10.1016/j.jenvrad.2018.09.017 30241014

